# Development and Validation of a Novel Nomogram Risk Prediction Model for In-Hospital Death Following Extended Aortic Arch Repair for Acute Type A Aortic Dissection

**DOI:** 10.31083/RCM26943

**Published:** 2025-04-21

**Authors:** Qiyi Chen, Yulin Wang, Yixiao Zhang, Fangyu Liu, Kejie Shao, Hao Lai, Chunsheng Wang, Qiang Ji

**Affiliations:** ^1^Department of Cardiovascular Surgery, Zhongshan Hospital Fudan University, 200032 Shanghai, China; ^2^Shanghai Municipal Institute for Cardiovascular Diseases, 200032 Shanghai, China

**Keywords:** acute type A aortic dissection, extended aortic arch repair, prediction model, machine learning, nomogram

## Abstract

**Background:**

Extended aortic arch repair (EAR) is increasingly adopted for treating acute type A aortic dissection (ATAAD). However, existing prediction models may not be suitable for assessing the in-hospital death risk in ATAAD patients undergoing EAR. This study aims to develop a comprehensive risk prediction model for in-hospital death following EAR based on patient’s preoperative status and surgical data, which may contribute to identification of high-risk individuals and improve outcomes following EAR.

**Methods:**

We reviewed clinical records of consecutive adult ATAAD patients undergoing EAR at our institute between January 2015 and December 2022. Utilizing data from 925 ATAAD patients undergoing EAR, we employed multivariable logistic regression and machine learning techniques, respectively, to develop nomograms for in-hospital mortality. Employed machine learning techniques included simple decision tree, random forest (RF), eXtreme Gradient Boosting (XGBoost), and support vector machine (SVM).

**Results:**

The nomogram based on SVM outperformed others, achieving a mean area under the receiver operating characteristic (ROC) curve (AUC) of 0.842 on training dataset and a mean AUC of 0.782 on testing dataset, accompanied by a Brier score of 0.058. Key risk factors included cerebral malperfusion, mesenteric malperfusion, preoperative critical station, Marfan syndrome, platelet count, D-dimer, coronary artery bypass grafting, and cardiopulmonary bypass time. A web-based application was developed for clinical use.

**Conclusions:**

We develop a novel nomogram risk prediction model based on SVM algorithm for in-hospital death following extended aortic arch repair for ATAAD with good discrimination and accuracy.

**Clinical Trial Registration:**

Registration number ChiCTR2200066414, https://www.chictr.org.cn/showproj.html?proj=187074.

## 1. Introduction

Acute type A aortic dissection (ATAAD), defined by an intimal tear and the dissection’s propagation between the media and intima layers of the aorta, remains one of the most challenging and highly morbid conditions encountered by cardiovascular surgeons. Despite advancements in surgical techniques and perioperative care, in-hospital mortality rates after ATAAD surgery remain significant, ranging from 5% to 20% in relatively stable patients, and reaching up to 35% in unstable cases [1, 2]. Extended aortic arch repair (EAR), which includes total arch replacement (TAR) combined with a frozen elephant trunk (FET), has gained popularity for its benefits in promoting favorable aortic remodeling, reducing the risk of reintervention, and enabling future completion of descending aortic procedures [3, 4]. Consequently, this approach is increasingly employed for managing ATAAD, particularly in China, where it has emerged as a preferred strategy [5]. However, EAR presents considerable challenges for cardiac surgeons, with its outcome being heavily dependent on the patient’s preoperative condition and the surgical components [6, 7]. Developing a risk prediction model that integrates preoperative characteristics and surgical data to estimate in-hospital mortality after EAR could aid in identifying high-risk patients, optimizing clinical decisions, and potentially improving surgical outcomes.

The widely used European System for Cardiac Operative Risk Evaluation (EuroSCORE) II [8], a standard tool in cardiovascular surgery, was not specifically designed for ATAAD patients and has been shown to have limited effectiveness in predicting ATAAD surgical outcomes [9]. The German Registry of Acute Aortic Dissection Type A (GERAADA) score, developed to estimate mortality risk in ATAAD surgery, may not be suitable for predicting outcomes in EAR procedures, as it is based on data from only 16% of patients who underwent the TAR procedure [6]. Additionally, the GERAADA score primarily considers preoperative risk factors, without including potentially significant intraoperative variables that could influence postoperative outcomes in ATAAD patients. The model developed by Rampoldi *et al*. [10], based on data from the International Registry of Acute Aortic Dissection in 2007, may also lack relevance due to advancements in surgical techniques over time. For example, only 11.5% of the patients included in their study underwent the TAR procedure [10]. Similarly, an early mortality prediction model for ATAAD repair developed by Zhang *et al*. [11] was limited by its small sample size and the potential exclusion of critical variables. These limitations suggest that current prediction models may not be well-suited for evaluating in-hospital death risk in ATAAD patients undergoing EAR. Moreover, the four models mentioned above were developed using logistic regression analysis, which is constrained by its assumption of linear relationships between predictors and outcomes. Machine learning offers an alternative approach, as it excels in identifying complex, non-linear patterns [12, 13]. Studies have demonstrated the potential of machine learning shines in analyzing the enormous data [14]. Thus, predictive models based on machine learning algorithms might be superior to those built using traditional logistic regression methods. However, the complexity of machine learning presents challenges in interpreting models and their outputs. Nomograms, known for their simplicity and utility in clinical practice, offer a promising solution. Incorporating variables identified through machine learning into a nomogram-based risk prediction model may be a promising approach to developing a risk prediction model. Nonetheless, data are scarce in this context.

In this study, we developed and validated prediction models using baseline characteristics and surgical data from consecutive ATAAD adult patients who underwent EAR at our institution between January 2015 and December 2022. The primary objective was to create a practical and accurate mortality risk prediction model by comparing the predictive performance and calibration of models constructed through logistic regression and machine learning techniques. We hypothesized that employing machine learning algorithms might provide superior predictive capability compared to traditional logistic regression methods.

## 2. Materials and Methods

### 2.1 Study Protocol and Study Population

This study was a single-center, retrospective analysis approved by the Institutional Review Board of our institution (No. B2022-592), with a waiver of individual consent. The study was registered with the Chinese Clinical Trial Registry (Registration number: ChiCTR2200066414, https://www.chictr.org.cn/showproj.html?proj=187074) and adhered to the Declaration of Helsinki. The research was conducted in compliance with the Strengthening the Reporting of Cohort, Cross-sectional and Case-control Studies in Surgery (STROCSS) criteria and aligned with the Transparent Reporting of a Multivariable Prediction Model for Individual prognosis or Diagnosis (TRIPOD) statement.

We reviewed the records of 925 adult ATAAD patients (aged >18 years) who underwent EAR, with or without additional procedures, at Zhongshan Hospital of Fudan University from January 2015 to December 2022. Exclusion criteria were as follows: (1) patients undergoing no-arch proximal aortic repair, hemiarch, or partial arch replacement; (2) patients undergoing fully endovascular or hybrid procedures, defined as a combination of surgical and endovascular approaches in the same setting; (3) cases of iatrogenic dissection; and (4) patients with dissection during pregnancy. No-arch proximal aortic repair, hemiarch, partial arch replacement, endovascular procedures, and hybrid procedures were excluded because these different surgical approaches could impact patient outcomes, and the study’s aim was to construct a predictive model specifically for EAR procedures. Including patients who underwent alternative surgical methods would not align with the study’s focus. Iatrogenic dissections were excluded due to their distinct onset mechanisms and management strategies, which might lead to varied clinical outcomes. Additionally, patients with dissection during pregnancy were excluded to account for the unique pathophysiological changes and mortality risks associated with pregnancy, such as amniotic fluid embolism.

### 2.2 Grouping

Eligible patients were categorized into two groups based on the occurrence of in-hospital death: the death group and the survival group. Baseline characteristics and surgical data were compared between these two groups.

### 2.3 Variable and Data

This study collected a comprehensive dataset comprising 108 clinical features. These included baseline characteristics (such as demographic, comorbidity, comorbidities, medical history, end-organ malperfusion, preoperative critical conditions, dissection characteristics, and laboratory and transthoracic echocardiographic [TTE] data), surgical details, and in-hospital outcomes. All data were retrieved from the hospital’s electronic database and reviewed using a standardized data collection form. Data collection was conducted by two trained staff members who were unaware of the study’s specific objectives to minimize bias. Discrepancies in data interpretation were resolved through consensus with a third reviewer. An independent database monitoring center was engaged to verify the plausibility of the dataset. Laboratory and TTE data were obtained within the first 24 hours before surgery. Only datasets that were validated through independent monitoring were included in the statistical analysis.

The primary outcome was in-hospital mortality, defined as all-cause deaths occurring within 30 days or any in-hospital deaths beyond 30 days for patients who had not been discharged after the index procedure. Previous cardiac surgery was defined as any prior major cardiac operation involving the opening of the pericardium [8]. A critical preoperative state was defined as the occurrence of one or more of the following occurring preoperatively in the same hospital admission as the operation: cardiac massage, preoperative ventilation prior to arrival in the anesthetic room, hypotension or shock, intra-aortic balloon counterpulsation, ventricular-assist device placement prior to arrival in the anesthetic room, or cardiac tamponade [8]. Malperfusion was defined as inadequate blood supply to specific organs due to aortic dissection, confirmed by clinical signs, symptoms, physical examination findings, and laboratory results [15]. Emergency surgery was defined as a procedure performed before the start of the next working day following the decision to operate [8]. Binary variables were encoded as 0 or 1 (0 = no, 1 = yes). Other categorical variables were preprocessed according to their nature. For example, the Neri classification of coronary involvement [16] was encoded as 0, 1, 2, or 3 (0 = no coronary involvement, 1 = Neri A class, 2 = Neri B class, and 3 = Neri C class). Similarly, the degree of aortic valve stenosis or regurgitation was encoded as 0, 1, 2, or 3 (0 = no stenosis/regurgitation, 1 = mild, 2 = moderate, and 3 = severe).

For handling missing data, the MissForest imputation method (R package “missForest”, https://CRAN.R-project.org/package=missForest) was employed to impute variables with less than 10% missing data. Variables with more than 10% missing data were excluded from the analysis. The distribution of variables before and after imputation was shown in **Supplementary Fig. 1**.

### 2.4 Development, Validation, and Comparison of Nomogram Prediction Models

For this study, the dataset was randomly divided into two subsets: a training dataset (70%) for model development and a testing dataset (30%) for model validation. Variables for the nomogram risk prediction models were identified using logistic regression analyses and machine learning techniques, including random forest (RF), decision tree (Dtree), eXtreme Gradient Boosting (XGBoost), and support vector machine (SVM). After variable selection, the optimal parameters for each nomogram risk prediction model were modified within the training dataset and subsequently validated using the testing dataset.

Nomogram risk prediction model 1 (Fit.logistic regression analysis (LR)) was constructed using univariate and multivariate binary logistic regression analyses to select variables. Variables with *p* < 0.05 in the between-group comparisons were included in the univariate logistic regression analysis performed on the training dataset. Subsequently, variables with *p* < 0.05 and odds ratios (OR) not equal to 1, as identified in the univariate analysis, were entered into the multivariate logistic regression analysis within the training dataset. Finally, variables with *p* < 0.05 and OR not equal to 1 from the multivariate analysis were incorporated into the Fit.LR model.

Four additional nomogram risk prediction models were constructed using machine learning algorithms for variable selection. Initially, four machine learning prediction models, RF, Dtree, XGBoost, and SVM, were developed on the training dataset. Variables with *p* < 0.05 in between-group comparisons were included in these models. To prevent overfitting, the machine learning models were constructed using the optimal subset of feature variables obtained via feature selection, selected to maximize model accuracy. The variations in prediction accuracy during the feature selection process are illustrated in **Supplementary Fig. 2**. The SHapley Additive exPlanations (SHAP) method [17] was applied to assess the significance of each variable in the machine learning models. To avoid overfitting in the nomogram risk prediction models, the principle of 10 events per variable (EPV) [18] was followed, as 84 valid events (in-hospital deaths) were observed in this cohort. Therefore, the top eight most significant variables were included in the final models. These variables were used to construct the nomograms, and the SHAP values for each variable in the machine learning models were shown in **Supplementary Fig. 3**.

The performance of the nomogram risk prediction models was evaluated by using receiver operating characteristic (ROC) curves, with the area under the ROC curve (AUC) calculated for all models. To ensure robust performance evaluation and to reduce the risk of overfitting, the AUC was computed using the bootstrap method, with 1000 resampling iterations. Nomograms were developed for models with good predictive performance to enhance practical applicability. Calibration curves were generated to assess the agreement between predicted and observed outcomes. The Brier score was used to quantify the difference between predicted and actual outcomes. Decision curve analysis (DCA) evaluated the utility of the predictive models in clinical decision-making by using net benefit as an indicator [19]. Additionally, the net reclassification index (NRI) and integrated discrimination improvement (IDI) were calculated to further evaluate the predictive accuracy and discriminatory ability of models with similar predictive performance. Finally, the nomogram prediction model demonstrating the best overall performance was integrated into a web-based survival calculator for ease of use.

### 2.5 Statistical Analysis

The incidence of in-hospital death in the cohort was estimated to be 10%. Based on a margin of error ≤0.05 and following established recommendations for developing clinical prediction models [20], the minimum required sample size was calculated to be 139 patients. To improve model stability and ensure the development of a more representative predictive model, the study included a total of 925 patients over an 8-year period.

The Shapiro-Wilks test was used to assess data normality. Continuous variables with a normal distribution were expressed as the mean ± standard deviation and compared between groups using the independent-sample *t*-test. Non-normally distributed continuous variables were reported as the median and interquartile range and compared using the Wilcoxon rank-sum test. Categorical variables were presented as frequencies and percentages and were compared between groups using the Chi-square test or Fisher’s exact test when the expected frequency was <5. To identify variables associated with in-hospital death, univariate and multivariate binary logistic regression analyses were conducted to calculate OR and 95% confidence intervals (CI). Statistical significance was set at *p* < 0.05 (two-sided). All statistical analyses were performed using SPSS version 26.0 (SPSS Inc., Chicago, IL, USA) and R version 4.3.2 (R Project for Statistical Computing, https://www.r-project.org).

### 2.6 Code Availability

R (version 4.3.2) was used for building and validating the predictive models. The complete code for this study was publicly available without restriction at the following repository: https://github.com/qiyi-chen/Nomogram-for-in-hospital-death-following-EAR.

## 3. Results

The variable distribution before and after imputation was shown in **Supplementary Fig. 1**, indicating no significant changes in data distribution following imputation. After random allocation, the distribution of variables in the training and testing datasets was presented in Table 1. As shown in Table 1, a statistically significant difference was observed in the time from symptom onset to surgery between the two datasets. However, this variable was not incorporated into the prediction models, and the overall distribution of variables in the training and testing datasets was considered balanced.

**Table 1.** **Characteristics of datasets**.

Variable			Training dataset	Testing dataset	*p*
			(N = 684)	(N = 277)	
Demographics					
	Male		519 (80.1%)	212 (76.5%)	0.223
	Age, years		52.0 (43.0–63.0)	52.0 (42.0–60.0)	0.255
	Height, cm		170.0 (166.0–175.0)	170.0 (165.0–175.0)	0.888
	Weight, kg		75.0 (65.0–83.0)	75.0 (65.0–83.0)	0.561
	BMI, kg/m^2^		25.6 (23.4–27.8)	25.3 (22.8–27.8)	0.339
	Somking		123 (19.0%)	45 (16.2%)	0.323
Comorbidity					
	HBP		470 (72.5%)	188 (67.9%)	0.152
	DM		31 (4.8%)	10 (3.6%)	0.427
	Stroke		31 (4.8%)	8 (2.9%)	0.189
	CAD		28 (4.3%)	12 (4.3%)	0.994
	CKD		15 (2.3%)	2 (0.7%)	0.115
	AF		10 (1.5%)	3 (1.1%)	0.765
	COPD		6 (0.9%)	1 (0.4%)	0.684
	AD family history		16 (2.5%)	6 (2.2%)	>0.999
	BAV		18 (2.8%)	8 (2.9%)	>0.999
	MFS		54 (8.3%)	23 (8.3%)	>0.999
	Heart surgery history		22 (3.4%)	12 (4.3%)	0.567
	Previous TEVAR		18 (2.8%)	9 (3.2%)	0.674
Medical history					
	Anticoagulation drugs		11 (1.7%)	3 (1.1%)	0.572
	Warfarin sodium		9 (1.4%)	3 (1.1%)	>0.999
	Rivaroxaban		2 (0.3%)	0	>0.999
	Antiplatelet drugs		19 (2.9%)	10 (3.6%)	0.681
	Aspirin		19 (2.9%)	10 (3.6%)	0.681
	Clopidogrel		9 (1.4%)	0	0.064
	Ticagrelor		0	0	>0.999
Malperfusion					
	IscCoronary		27 (4.2%)	7 (2.5%)	0.257
	IscCerebral		67 (10.3%)	24 (8.7%)	0.433
	IscSpinal		14 (2.2%)	4 (1.4%)	0.607
	IscMesenteric		16 (2.5%)	8 (2.9%)	0.822
	IscRenal		48 (7.4%)	25 (9.0%)	0.403
	IscUEM		12 (1.9%)	1 (0.4%)	0.123
	IscLEM		76 (11.7%)	31 (11.2%)	0.815
Critical preoperative status			62 (9.6%)	31 (11.2%)	0.452
	Hypotension		21 (3.2%)	15 (5.4%)	0.117
	Shock		4 (0.6%)	2 (0.7%)	>0.999
	Tamponade		19 (2.9%)	14 (5.1%)	0.123
	Ventilation		22 (3.4%)	10 (3.6%)	0.846
Laboratory data					
	Hb, g/L		133.0 (122.0–145.0)	132.0 (120.5–144.0)	0.354
	WBC, ×10^12^/L		12.0 (9.8–14.6)	11.9 (10.0–15.0)	0.607
	Plt, ×10^9^/L		155.0 (124.0–194.0)	161.0 (134.4–201.0)	0.074
	N, ×10^12^/L		10.2 (8.0–12.5)	10.0 (8.0–12.9)	0.600
	cTnT, ×1000 ng/mL		20.0 (10.0–68.8)	20.0 (9.0–86.2)	0.685
	BNP, pg/mL		317.3 (144.1–763.2)	348.2 (162.0–777.1)	0.614
	Fibrinogen, mg/dL		243.5 (184.0–377.0)	253.0 (182.5–380.0)	0.534
	D2, mg/L		8.8 (4.0–15.7)	8.8 (3.9–14.7)	0.660
	INR		1.1 (1.0–1.1)	1.1 (1.0–1.2)	0.125
	Tbil, µmol/L		16.5 (12.3–22.8)	16.3 (11.8–21.5)	0.536
	Albumin, g/L		40.0 (37.0–43.0)	40.0 (37.0–43.0)	0.436
	ALT, U/L		25.0 (17.0–44.0)	28.0 (16.0–46.0)	0.533
	AST, U/L		25.0 (18.0–40.1)	26.0 (17.5–44.7)	0.473
	Urea, mmol/L		6.7 (5.4–8.9)	7.0 (5.4–8.9)	0.753
	Cr, µmol/L		87.0 (70.0–115.0)	83.0 (66.0–117.0)	0.306
	Na, mmol/L		140.0 (138.0–142.0)	139.0 (137.8–142.0)	0.677
	K, mmol/L		3.8 (3.5–4.1)	3.8 (3.6–4.1)	0.656
TTE data					
	Root, mm		40.0 (36.0–44.0)	40.0 (36.0–44.0)	0.861
	LAD, mm		36.2 (33.0–39.0)	36.0 (33.0–39.0)	0.164
	LVEDD, mm		48.0 (44.2–51.0)	48.0 (45.0–51.0)	0.536
	LVESD, mm		30.6 (29.0–33.0)	31.0 (29.0–33.0)	0.424
	IVS, mm		11.4 (10.0–12.3)	11.0 (10.0–12.2)	0.166
	LVEF, %		63.2 (61.0–66.0)	64.0 (61.0–66.0)	0.880
	ProxAo, mm		44.0 (41.0–49.0)	45.0 (41.0–50.0)	0.364
	AI				0.128
		No or trace	256 (39.5%)	103 (37.2%)	
		Mild	154 (23.8%)	75 (27.1%)	
		Moderate	145 (22.4%)	46 (16.6%)	
		Moderate to sever	60 (9.3%)	33 (11.9%)	
		Severe	33 (5.1%)	20 (7.2%)	
	AS				0.459
		No or trace	642 (99.1%)	834 (99.2%)	
		Mild	1 (0.2%)	2 (0.7%)	
		Moderate	4 (0.6%)	2 (0.7%)	
		Moderate to sever	1 (0.2%)	0	
		Severe	0	0	
	Pericardial effusion		197 (30.4%)	93 (33.6%)	0.341
Characteristics of dissection					
	IMH		64 (9.9%)	34 (12.3%)	0.278
	PAU		14 (2.2%)	6 (2.2%)	>0.999
	Thrombosis of the false lumen				
		Root	68 (10.5%)	27 (9.7%)	0.732
		Ascending	151 (23.3%)	77 (27.8%)	0.146
		Arch	69 (10.6%)	29 (10.5%)	0.935
		Descending	18 (2.8%)	2 (0.7%)	0.050
	Entry tear				
		Root	22 (3.4%)	10 (3.6%)	0.846
		Ascending	310 (47.8%)	152 (54.9%)	0.050
		Arch	30 (35.7%)	283 (33.7%)	0.076
		Descending	156 (24.1%)	61 (22.0%)	0.500
	Commissure detachment		307 (47.4%)	126 (45.5%)	0.598
	Sinus involved		427 (65.9%)	180 (65.0%)	0.789
	Coronary involvement				
	RCA				0.804
		None	480 (74.1%)	209 (75.5%)	
		Neri A	57 (8.8%)	19 (6.9%)	
		Neri B	96 (14.8%)	42 (15.2%)	
		Neri C	15 (2.3%)	7 (2.5%)	
	LCA				0.618
		None	619 (95.5%)	262 (94.6%)	
		Neri A	11 (1.7%)	5 (1.8%)	
		Neri B	16 (2.5%)	10 (3.6%)	
		Neri C	2 (0.3%)	0	
	Supra-aortic vessels involvement				
		IA	407 (62.8%)	183 (66.1%)	0.345
		LCCA	294 (45.4%)	135 (48.7%)	0.347
		LSCA	280 (43.2%)	138 (49.8%)	0.064
Duration					
	Sym.hosT, h		13.0 (7.0–24.0)	15.0 (8.0–26.5)	0.116
	Hos.surgT, h		13.0 (5.0–24.0)	15.0 (6.0–24.0)	0.115
	Sym.surgT, h		28.0 (17.0–63.8)	32.0 (20.0–71.0)	0.042
	Emergency		506 (78.1%)	210 (75.8%)	0.449
Surgical data					
Proximal					
	ARR		455 (70.2%)	177 (63.9%)	0.059
	Bentall		66 (10.2%)	38 (13.7%)	0.119
	David		58 (9.0%)	33 (11.9%)	0.166
	Wheat		2 (0.3%)	3 (1.1%)	0.162
Distal					
	TAR		684 (100%)	277 (100%)	>0.999
	FET		639 (98.6%)	269 (97.1%)	0.120
Associated surgeries					
	CABG		44 (6.8%)	24 (8.7%)	0.317
	Other				/
		MV procedures	4 (0.6%)	1 (0.4%)	>0.999
		TV procedures	0	0	>0.999
Perfusion					
	CPB time, min		185.0 (160.0–214.0)	187.0 (162.0–220.0)	0.268
		Re-CPB	16 (2.5%)	8 (2.9%)	0.822
		Re-re-CPB	3 (0.5%)	1 (0.4%)	>0.999
	ACC, min		103.0 (84.0–127.0)	107.0 (86.5–129.0)	0.135
	Re-ACC		8 (1.2%)	4 (1.4%)	0.759
	DHCA, min		21.0 (17.0–26.0)	20.0 (17.0–27.0)	0.984
		Unilateral ACP	644 (99.4%)	273 (98.6%)	0.214
		Bilateral ACP	17 (2.6%)	4 (1.4%)	0.341
	LNT, °C		22.0 (21.0–23.0)	22.1 (21.1–23.0)	0.229
	LBT, °C		25.9 (25.0–26.8)	26.0 (25.1–27.0)	0.063
Blood product					
	Transfusion rate		503 (77.6%)	220 (79.4%)	0.544
	Red cell, U		7.5 (4.0–10.0)	4.0 (2.0–6.0)	0.566
	Plam, mL		800.0 (600.0–1200.0)	600.0 (5.0–800.0)	0.934
	Mortality		60 (9.3%)	24 (8.7%)	0.773

BMI, body mass index; HBP, high blood pressure; DM, diabetes mellitus; CAD, coronary artery disease; CKD, chronic kidney disease; AF, atrial fibrillation; COPD, chronic obstructive pulmonary disease; AD, aortic dissection; MFS, Marfan syndrome; BAV, bicuspid aortic valve; TEVAR, thoracic endovascular aortic repair; IscCerebral, cerebral malperfusion; IscSpinal, spinal malperfusion; IscCoronary, coronary malperfusion; IscMesenteric, mesenteric malperfusion; IscRenal, renal malperfusion; IscUEM, upper extremity malperfusion; IscLEM, lower extremity malperfusion; Hb, hemoglobin; WBC, white blood cell count; Plt, platelet count; N, neutrophil count; BNP, brain natriuretic peptide; D2, D-dimer; INR, international normalized ratio; ALT, alanine transaminase; AST, aspartate aminotransferase; Cr, creatinine; LAD, diameter of left atrium; LVEDD, left ventricular end-diastolic dimension; LVESD, left ventricular end-systolic dimension; IVS, interventricular septum; LVEF, left ventricular ejection fraction; ProxAo, diameter of the ascending aorta; AI, aortic insufficiency; AS, aortic valve stenosis; IMH, intramural hematoma; PAU, penetration aortic ulcer; Sym.hosT, time to hospital from symptom onset; Hos.surgT, time to surgery from hospital onset; Sym.surgT, time to surgery from symptom onset; RCA, right coronary artery; LCA, left coronary artery; IA, innominate artery; LCCA, left common carotid artery; LSCA, left subclavian artery; ARR, ascending aorta replacement with commissure resuspension; CABG, coronary artery bypass grafting; MV procedures, mitral valve procedures; TV procedures, tricuspid valve procedures; FET, frozen elephant trunk; CPB, cardiopulmonary bypass; ACC, aortic cross-clamp time; DHCA, deep hypothermic circulatory arrest; ACP, anterograde cerebral perfusion; LNT, lowest nose temperature; LBT, lowest bladder temperature; Reb cell, intraoperative red blood cell transfusions; Plam, intraoperative plasma transfusions; cTnT, cardiac troponin T; TTE, transthoracic echocardiographic; TAR, total arch replacement; Tbil, total bilirubin.

### 3.1 Study Population and Risk Factors for in-Hospital Death

**Fig. 1.**
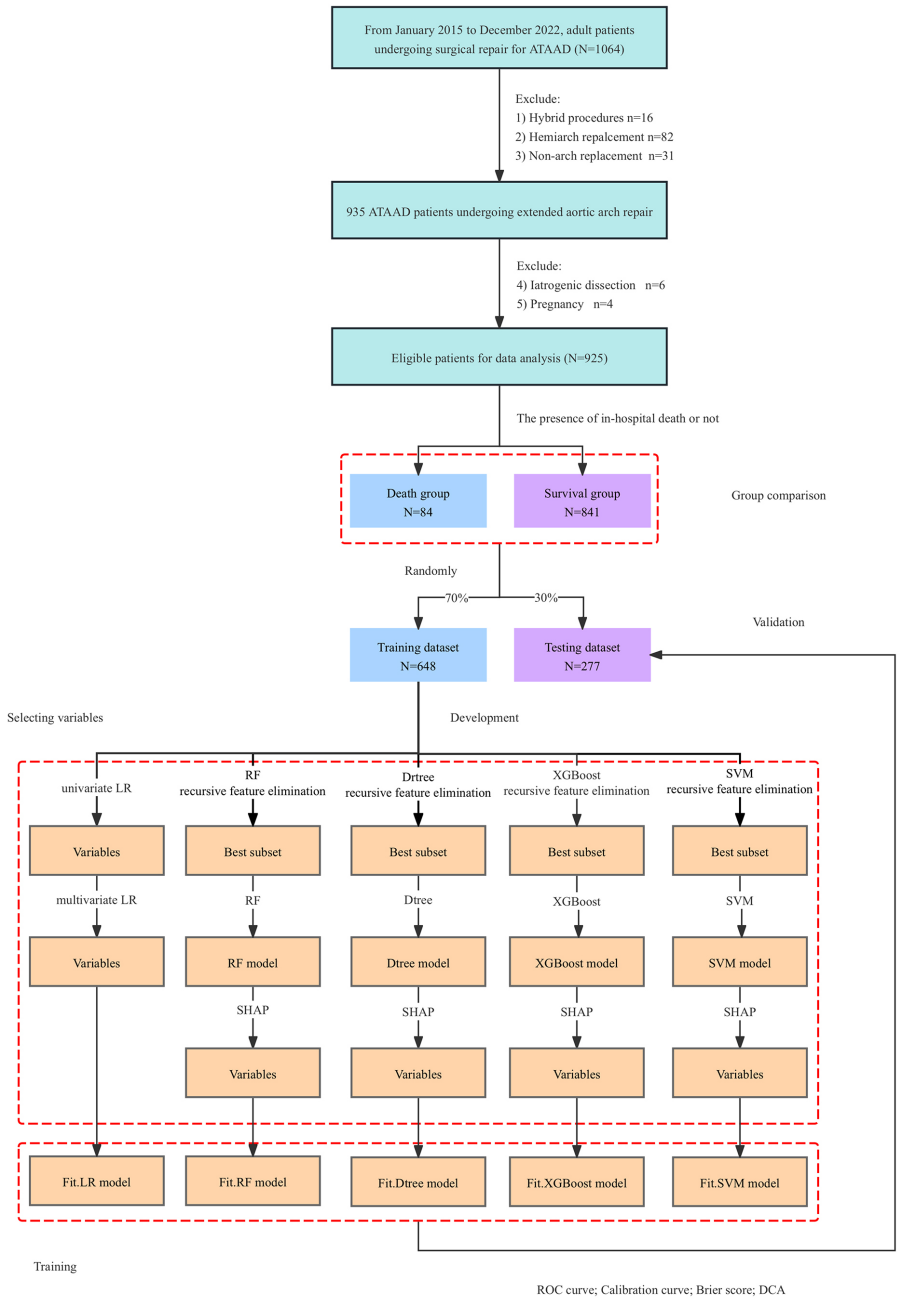
**Flow chart for the selection of study population and study design**. ATAAD, acute type A aortic dissection; FET, frozen elephant trunk; LR, logistic regression analysis; RF, random forest; Dtree, decision tree; XGBoost, eXtreme Gradient Boosting; SVM, support vector machine; SHAP, SHapley Additive exPlanations; ROC, receiver operating characteristic; DCA, decision curve analysis.

A total of 1064 adult patients underwent surgical repair of ATAAD at our center over an 8-year period. After excluding 139 patients (Fig. 1), 925 eligible patients were included in the analysis and categorized into the death group (n = 84) or the survival group (n = 841). Among the study population, the average age was 51.9 ± 12.4 years, and the mean body mass index (BMI) was 25.7 ± 3.7 kg/m^2^, with 731 (79%) patients being male. Significant differences in baseline characteristics and surgical data were observed between the two groups, as detailed in **Supplementary Table 1**.

The results of univariate and multivariate logistic regression analyses on the training dataset are presented in Table 2. After conducting both univariate and multivariate logistic regression analyses, the following variables were identified as independent predictors of in-hospital death following EAR for ATAAD: cerebral malperfusion, mesenteric malperfusion, critical preoperative status (CPStatus), D-dimer (D2), international normalized ratio (INR), cardiopulmonary bypass (CPB) time, and coronary artery bypass grafting (CABG).

**Table 2.** **Univariable and Multivariable logistic regression analyses on the training set**.

Variable		Univariable OR (95% CI, *p*)	Multivariate OR (95% CI, *p*)
MFS		0.17 (0.02–1.26, *p* = 0.083)	
IscCoronary		5.59 (2.39–13.07, *p* < 0.001)	1.22 (0.31–4.29, *p* = 0.800)
*IscCerebral		4.71 (2.52–8.81, *p* < 0.001)	2.77 (1.10–6.63, *p* = 0.025)
IscSpinal		5.85 (1.89–18.06, *p* = 0.002)	3.06 (0.73–11.60, *p* = 0.110)
*IscMesenteric		19.40 (6.77–55.58, *p* < 0.001)	10.1 (2.32–44.40, *p* = 0.002)
IscRenal		3.34 (1.61–6.96, *p* = 0.001)	1.19 (0.40–3.28, *p* = 0.700)
IscLEM		2.58 (1.34–4.96, *p* = 0.004)	2.33 (0.92–5.58, *p* = 0.064)
*Critical preoperative status		7.93 (4.29–14.68, *p* < 0.001)	4.65 (1.22–17.10, *p* = 0.022)
Hypotension		4.24 (1.58–11.39, *p* = 0.004)	0.49 (0.04–4.70, *p* = 0.600)
Shock		10.10 (1.40–73.06, *p* = 0.022)	0.41 (0.01–20.50, *p* = 0.700)
Tamponade		4.91 (1.80–13.45, *p* = 0.002)	1.16 (0.10–14.80, *p* > 0.999)
Ventilation		7.81 (3.18–19.14, *p* < 0.001)	1.22 (0.32–4.50, *p* = 0.800)
WBC, ×10^12^/L		1.08 (1.01–1.16, *p* = 0.018)	1.18 (0.74–1.85, *p* = 0.500)
Plt, ×10^9^/L		0.99 (0.99–1.00, *p* = 0.004)	0.99 (0.99–1.00, *p* = 0.110)
N, ×10^12^/L		1.09 (1.01–1.17, *p* = 0.018)	0.79 (0.49–1.28, *p* = 0.300)
cTnT, ×1000 ng/mL		1.07 (1.00–1.15, *p* = 0.064)	
BNP, pg/mL		1.00 (1.00–1.00, *p* = 0.023)	
Fibrinogen, mg/dL		1.00 (0.99–1.00, *p* = 0.001)	
*D2, mg/L		1.06 (1.03–1.08, *p* < 0.001)	1.03 (1.00–1.07, *p* = 0.034)
*INR		4.56 (2.01–10.38, *p* < 0.001)	3.48 (1.12–9.41, *p* = 0.016)
Albumin, g/L		0.96 (0.91–1.01, *p* = 0.096)	
AST, U/L		1.00 (1.00–1.00, *p* = 0.018)	
Urea, mmol/L		1.00 (0.99–1.02, *p* = 0.734)	
Cr, µmol/L		1.00 (1.00–1.00, *p* = 0.037)	
IVS, mm		1.17 (1.01–1.34, *p* = 0.030)	1.14 (0.94–1.38, *p* = 0.200)
AS			
	no or trace	\	
	mild	0.00 (0.00–Inf, *p* = 0.993)	
	moderate	0.00 (0.00–Inf, *p* = 0.985)	
	moderate to sever	56894998.27 (0.00–Inf, *p* = 0.990)	
	severe	\	
Thrombosis of the false lumen of aortic root		0.13 (0.02–0.97, *p* = 0.046)	0.20 (0.01–1.07, *p* = 0.130)
*CABG		6.43 (3.21–12.85, *p* < 0.001)	4.01 (1.44–10.70, *p* = 0.006)
*CPB time, min		1.01 (1.01–1.02, *p* < 0.001)	1.01 (1.00–1.02, *p* < 0.001)
	Re-re-CPB	20.24 (1.81–226.62, *p* = 0.015)	1.58 (0.09–50.10, *p* = 0.800)
ACC, min		1.01 (1.00–1.01, *p* = 0.054)	
	Bilateral ACP	2.16 (0.60–7.73, *p* = 0.238)	
Transfusion rate		2.79 (1.17–6.61, *p* = 0.020)	1.04 (0.30–4.06, *p* > 0.999)
Red cell, U		1.12 (1.06–1.19, *p* < 0.001)	1.04 (0.93–1.15, *p* = 0.500)
Plam, mL		1.00 (1.00–1.00, *p* < 0.001)	

OR, odds ratio; CI, confidence interval; MFS, Marfan syndrome; IscCerebral, cerebral malperfusion; IscSpinal, spinal malperfusion; IscCoronary, coronary malperfusion; IscMesenteric, mesenteric malperfusion; IscRenal, renal malperfusion; IscLEM, lower extremity malperfusion; WBC, white blood cell count; Plt, platelet count; N, neutrophil count; BNP, n-terminal pro-brain natriuretic peptide; D2, D-dimer; INR, international normalized ratio; AST, aspartate aminotransferase; Cr, creatinine; IVS, interventricular septum; AS, aortic valve stenosis; CABG, coronary artery bypass grafting; CPB, cardiopulmonary bypass; ACC, aortic cross-clamp time; ACP, anterograde cerebral perfusion; Reb, intraoperative red blood cell transfusions; Plam, intraoperative plasma transfusions; cTnT, cardiac troponin T; Inf, infinity. * Variables were finally included in the Fit.LR model.

### 3.2 Variables of Nomogram Risk Prediction Models

The following variables were included in the Fit.LR model after logistic regression analyses: cerebral malperfusion, mesenteric malperfusion, CPStatus, D2, INR, CPB time, and CABG. The Fit.RF model incorporated eight variables: mesenteric malperfusion, cardiac tamponade, D2, INR, platelet count (Plt), albumin levels, CPB time, and intraoperative red blood cell transfusion. For the Fit-Dtree model, the eight variables included were: mesenteric malperfusion, hypotension, Marfan syndrome (MFS), Plt, cardiac troponin T (cTnT), INR, D2, and CPB time. The Fit.XGBoost model utilized the following variables: mesenteric malperfusion, CPStatus, Plt, D2, aspartate aminotransferase (AST), creatinine (Cr), CPB time, and aortic cross-clamp time (ACC). Lastly, the Fit.SVM model analyzed these variables: cerebral malperfusion, mesenteric malperfusion, CPStatus, D2, Plt, CABG, intraoperative blood product transfusion, and CPB time.

### 3.3 Performances of Nomogram Risk Prediction Models

**Fig. 2.**
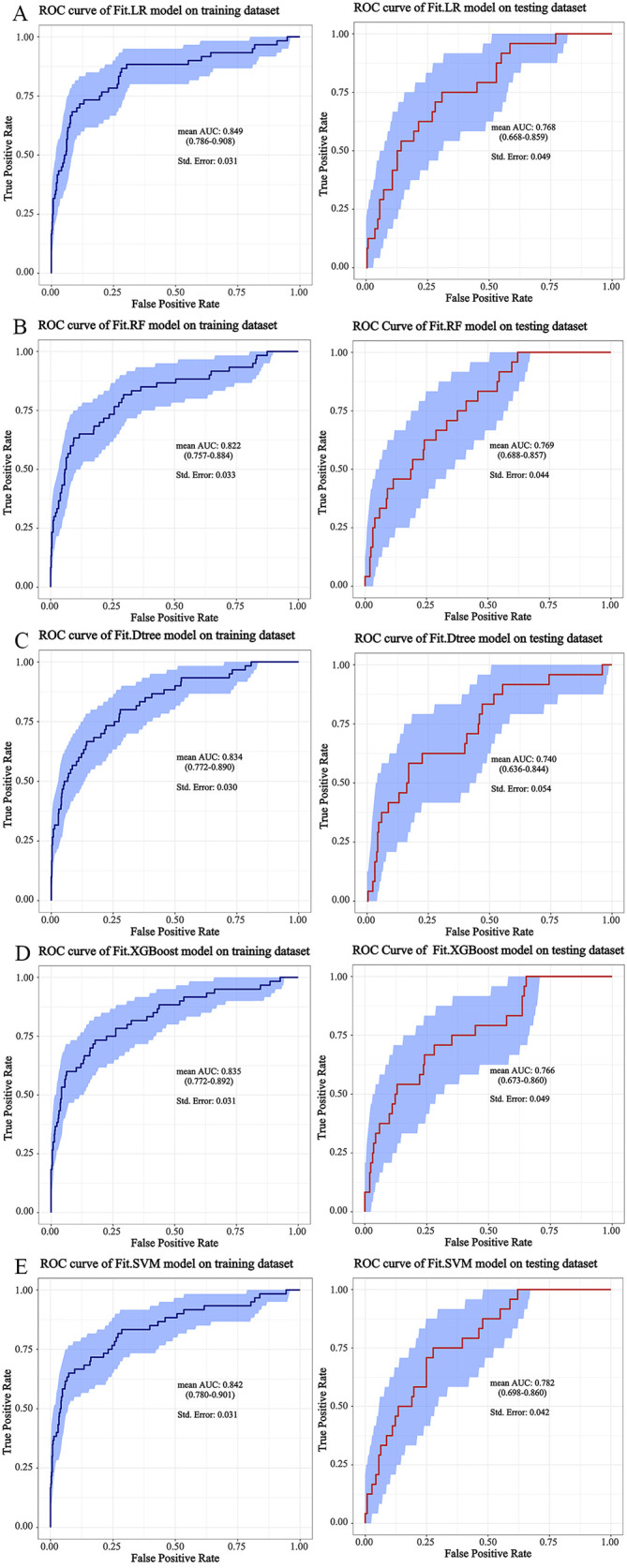
**The ROC curves of models**. (A) The ROC curves of Fit.LR model; (B) the ROC curves of Fit.RF model; (C) the ROC curves of Fit.Dtree model; (D) the ROC curves of Fit.XGBoost model; (E) the ROC curves of Fit.SVM model. ROC, receiver operating characteristic; LR, logistic regression analysis; RF, random forest; Dtree, decision tree; XGBoost, eXtreme Gradient Boosting; SVM, support vector machine; AUC, the area under the receiver operating characteristic curve.

**Fig. 3.**
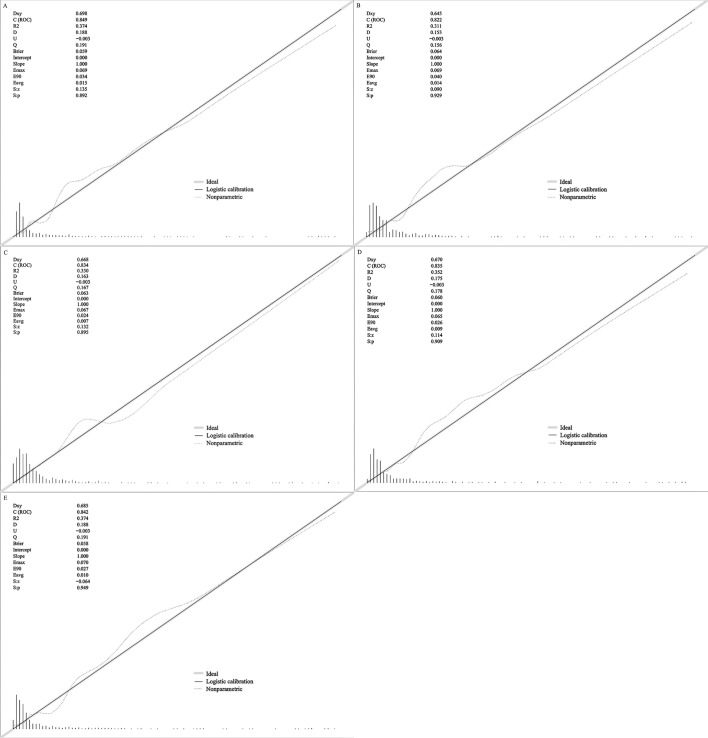
**The calibration curves of models**. (A) The calibration curves of Fit.LR model; (B) the calibration curves of Fit.RF model; (C) the calibration curves of Fit.Dtree model; (D) the calibration curves of Fit.XGBoost model; (E) the calibration curves of Fit.SVM model. ROC, receiver operating characteristic; R2, coefficient of complex determination; D, discrimination index; U, unreliability index; Q, quality index; LR, logistic regression analysis; RF, random forest; Dtree, decision tree; XGBoost, eXtreme Gradient Boosting; SVM, support vector machine; Dxy, the magnitude of the rank correlation between the predicted probability and the observed value; Emax, the maximum absolute difference between the predicted value and the actual value; E90, the 90th percentile of the difference between the predicted value and the true value; Eavg, the average difference between the predicted value and the actual value; S:z, Z-value of Spiegelhalter Z-test; S:p, *p*-value of Spiegelhalter Z-test.

**Fig. 4.**
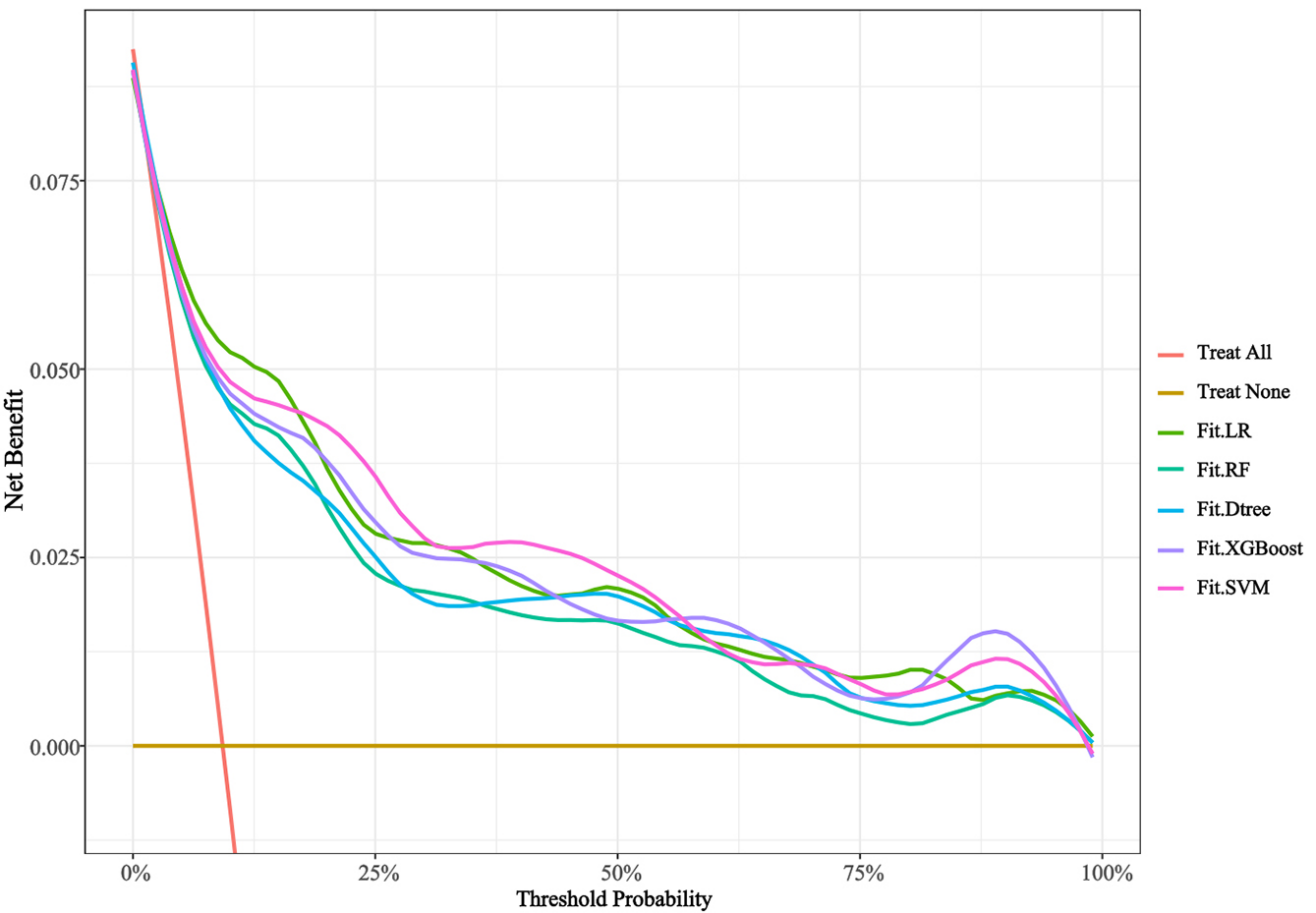
**The DCA curves of models**. LR, logistic regression analysis; RF, random forest; Dtree, decision tree; XGBoost, eXtreme Gradient Boosting; SVM, support vector machine; DCA, decision curve analysis.

The predictive performances of all five models were evaluated using both the training and testing datasets. As shown in Table 3 and Fig. 2, the Fit.LR model demonstrated the highest mean AUC value on the training dataset (0.849, 95% CI 0.786 to 0.908), followed by the Fit.SVM model (0.842, 95% CI 0.780 to 0.910), the Fit.XGBoost model (0.835, 95% CI 0.772 to 0.892), the Fit.Dtree model (0.834, 95% CI 0.772 to 0.890), and the Fit.RF model (0.822, 95% CI 0.757 to 0.884). On the testing dataset, the Fit.SVM model achieved the highest mean AUC value (0.782, 95% CI 0.698 to 0.860), followed by the Fit.RF model (0.769, 95% CI 0.688 to 0.857), the Fit.LR model (0.768, 95% CI 0.668 to 0.859), the Fit.XGBoost model (0.766, 95% CI 0.673 to 0.860), and the Fit.Dtree model (0.740, 95% CI 0.636 to 0.860). Among the models, the Fit.Dtree model had the lowest standard error (0.030), followed by the Fit.LR model (0.031), the Fit.SVM model (0.031), the Fit.XGBoost model (0.031), and the Fit.RF model (0.033). Calibration curves showed that the predicted probabilities for all five models were comparable to the actual observations (Fig. 3). All models demonstrated a good fit based on the Hosmer-Lemeshow test. Additionally, the Fit.SVM model had the lowest Brier score (0.058), followed by the Fit.LR model (0.059), the Fit.XGBoost model (0.060), the Fit.Dtree model (0.063), and the Fit.RF model (0.064), indicating effective probability calibration. The DCA curves for each prediction model are presented in Fig. 4. As shown, all five models outperformed the “treat all” and “treat none” strategies across the risk threshold range of 1.8% to 100%, suggesting considerable clinical utility for all models. Among them, the Fit.SVM model exhibited the largest area under the curve, indicating its superior performance across various decision-making scenarios.

**Table 3.** **Performance of the 5 models**.

Models	Mean AUC on training set	Mean AUC on testing set	*p* ^*^	Brier score	Std. Error
Fit.LR	0.849 (95% CI 0.786 to 0.908)	0.768 (95% CI 0.668 to 0.859)	0.892	0.059	0.031
Fit.RF	0.822 (95% CI 0.757 to 0.884)	0.769 (95% CI 0.688 to 0.857)	0.929	0.064	0.033
Fit.Dtree	0.834 (95% CI 0.772 to 0.890)	0.740 (95% CI 0.636 to 0.844)	0.895	0.063	0.030
Fit.XGBoost	0.835 (95% CI 0.772 to 0.892)	0.766 (95% CI 0.673 to 0.860)	0.909	0.060	0.031
Fit.SVM	0.842 (95% CI 0.780 to 0.901)	0.782 (95% CI 0.698 to 0.860)	0.949	0.058	0.031

AUC, the area under the receiver operating characteristic curve; Std. Error, standard error; CI, confidence interval; LR, logistic regression analysis; RF, random forest; Dtree, decision tree; XGBoost, eXtreme Gradient Boosting; SVM, support vector machine. ^*^, *p* value for HosmerLemeshow test.

### 3.4 Comparison of Nomogram Risk Prediction Models

**Fig. 5.**
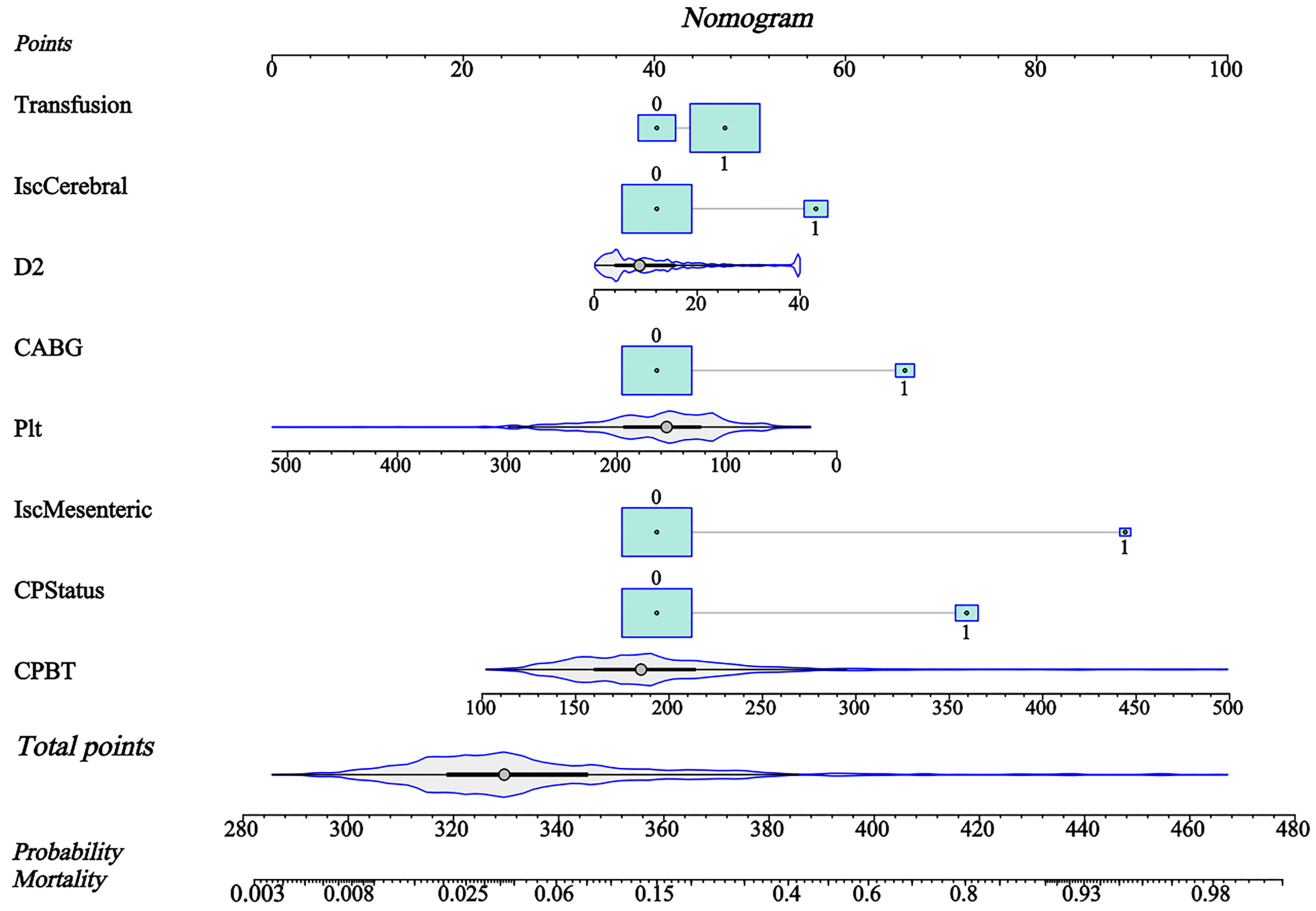
**The nomogram of Fit.SVM**. IscCerebral, cerebral malperfusion; D2, D-dimer; Plt, platelet count; CABG, coronary artery bypass grafting; IscMesenteric, mesenteric malperfusion; CPStatus, critical preoperative status; Transfusion, intraoperative blood product transfusion; CPBT, cardiopulmonary bypass time; SVM, support vector machine.

In summary, the Fit.SVM model outperformed other predictive models based on machine learning algorithms. It achieved the highest AUC values on both the training and testing datasets while demonstrating minimal deviation between predicted results and actual outcomes, as reflected in its lowest Brier score. Consequently, the Fit.SVM model was further compared with the Fit.LR model, which was constructed using logistic regression. Both the Fit.LR and Fit.SVM models showed excellent predictive performance. To further compare these models, the NRI and IDI were calculated. Using the Fit.LR model as the baseline and the Fit.SVM model as the comparator, in-hospital mortality <20% was classified as low risk, while mortality ≥20% was classified as high risk. The Fit.SVM model improved prediction accuracy by 14.3% (NRI 0.143, 95% CI 0.030 to 0.257, *p* = 0.013) and enhanced overall predictive ability by 6.2% (IDI 0.062, 95% CI 0.020 to 0.104, *p* = 0.004) compared to the Fit.LR model. These results demonstrated that the Fit.SVM model provided superior predictive performance. A nomogram was developed based on the Fit.SVM model to estimate the probability of a composite endpoint event (Fig. 5). The equation for the Fit.SVM model is as follows: Fit.SVM model = –5.040871 + (0.886100 × cerebral malperfusion) + (2.608367 × mesenteric malperfusion) + (1.725816 × CPStatus) + (0.028642 × D2) + (–0.006118 × Plt) + (1.381637 × CABG) + (0.379383 × intraoperative blood product transfusion) + (0.010404 × CPB time). Additionally, a web-based survival calculator based on the Fit.SVM model has been created and can be accessed at: https://heartsugery7zs-hospital.shinyapps.io/DynNomapp/.

### 3.5 Stratified Analyses

Patients were stratified into low-risk and high-risk groups based on the probability of death predicted by the Fit.SVM model, with a threshold of 20%. As shown in Table 4, 810 low-risk patients were identified in the cohort, with 37 in-hospital deaths, corresponding to a mortality rate of 4.6%. Meanwhile, 115 high-risk patients were identified, of whom 47 died during hospitalization, resulting in a mortality rate of 40.8%. The difference in the incidence of in-hospital death between the low-risk and high-risk groups was statistically significant. The distribution of variables included in the Fit.SVM model also differed significantly between the low-risk and high-risk groups. Notably, variables with a substantial impact on mortality, such as cerebral malperfusion (univariate OR 4.71, 95% CI 2.52 to 8.81, *p* < 0.001), mesenteric malperfusion (univariate OR 19.40, 95% CI 6.77 to 55.58, *p* < 0.001), and CPStatus (univariate OR 7.93, 95% CI 4.29 to 14.68, *p* < 0.001), were predominantly observed in the high-risk group.

**Table 4.** **Characteristics of low risk group and high risk group**.

Variables	Low risk group	High risk group	*p*
	(N = 810)	(N = 115)	
IscCerebral	55 (6.8%)	36 (31.3%)	<0.001
IscMesenteric	2 (0.2%)	22 (19.1%)	<0.001
Critical preoperative status	34 (4.2%)	59 (51.8%)	<0.001
D2	7.7 (3.7–13.8)	18.1 (9.6–32.0)	<0.001
Plt	159.0 (130.0–198.3)	134.7 (102.0–180.0)	<0.001
CABG	26 (3.2%)	42 (36.5%)	<0.001
Transfusion	620 (76.5%)	103 (89.5%)	0.002
CPBT	182.5 (158.0–210.0)	231.0 (179.0–300.0)	<0.001
Mortality	37 (4.6%)	47 (40.8%)	<0.001

IscCerebral, cerebral malperfusion; IscMesenteric, mesenteric malperfusion; D2, D-dimer; Plt, platelet count; CABG, coronary artery bypass grafting; CPBT, cardiopulmonary bypass time; Transfusion, intraoperative blood product transfusion.

## 4. Discussion

In this cohort of 925 adult patients who underwent EAR for ATAAD over an 8-year period, the in-hospital mortality rate was 9.1%, aligning with findings from previous studies [1, 2]. EAR has become a widely adopted surgical approach in China for ATAAD involving the aortic arch and descending thoracic aorta, with an acceptable in-hospital mortality rate [6, 7]. In recent years, EAR has attracted significant attention and is increasingly utilized in clinical practice. Current guidelines also recommend EAR as a surgical strategy for treating ATAAD [15]. EAR, however, has been considered to be the most difficult and challenging among all kinds of surgical procedures for ATAAD with a high risk for mortality [21]. Identifying high-risk patients and improving outcomes following EAR requires the development of a practical and effective risk prediction model for in-hospital death. In this study, we developed five nomogram models based on different methods for selecting predictive variables to estimate the risk of in-hospital mortality after EAR for ATAAD. Among these, the Fit.SVM model, constructed using the SVM machine learning algorithm, demonstrated excellent predictive performance on both the training and testing datasets. It also showed strong discrimination and calibration capabilities.

In this study, the SHAP method [17] was utilized to evaluate the significance of each variable in the machine learning model, aiding in variable selection through machine learning algorithms. SHAP serves as a robust tool that visualizes the predictions of the final model, making it widely recognized for improving the interpretability of machine learning models. By offering a unified framework, SHAP quantifies the individual contributions of each feature to the prediction, whether positive or negative, thereby enhancing the model’s explainability and transparency [22]. Overfitting, a common issue in machine learning, can undermine the predictive accuracy of models [23, 24]. It typically arises when a model becomes overly complex [25], resulting in erroneous conclusions that may lead to inappropriate clinical decisions. To counteract overfitting, strategies such as reducing noise (irrelevant data), feature selection, early stopping, and k-fold cross-validation are often employed [25, 26]. In this study, variables with *p* < 0.05 between the death and survival groups were selected to minimize irrelevant data. Additionally, the optimal subset of features identified through feature selection was used to reduce the risk of overfitting. Despite these precautions, the possibility of overfitting cannot be entirely excluded, and it may have implications for clinical outcomes.

Through univariable and multivariable logistic regression analyses, cerebral malperfusion, mesenteric malperfusion, CPStatus, D2, INR, CPB time, and the need for CABG were identified as independent risk factors for in-hospital death following EAR. Using the SVM algorithm, eight variables from the 108 variables analyzed were selected and incorporated into the Fit.SVM model: cerebral malperfusion, mesenteric malperfusion, CPStatus, D2, Plt, CABG, intraoperative blood product transfusion, and CPB time. The strong predictive performance of the Fit.SVM model, demonstrated by the ROC curve, calibration curve, and DCA, suggests that the selected combination of variables is well-suited for forecasting outcomes in ATAAD patients. SVM, a machine learning method with exceptional classification and generalization capabilities [27], proved to be an effective tool in this context. Additionally, machine learning algorithms not only identified variables significantly associated with mortality in univariate analysis but also uncovered variables that lacked statistical significance in univariate logistic regression. This highlights the advantages of machine learning over logistic regression, particularly in capturing non-linear relationships between variables and outcomes [12, 13]. Compared to the Fit.LR model, which was based on logistic regression analysis, the Fit.SVM model demonstrated superior prediction accuracy and overall predictive capability. These findings highlight the potential of machine learning algorithms in selecting variables and constructing nomogram-based risk prediction models.

Factors influencing in-hospital outcomes of ATAAD repair have been extensively documented in the literature [28, 29]. Previous studies have indicated that ATAAD patients presenting with cardiac tamponade, shock, congestive heart failure, cerebrovascular accident, stroke, coma, cerebral malperfusion, coronary malperfusion, or mesenteric malperfusion are classified as unstable and have an in-hospital mortality rate of 35%, compared to stable patients [1, 2]. In this study, cerebral malperfusion, mesenteric malperfusion, CPStatus, D2, INR, CPB time, and the need for CABG were identified as independent risk factors for in-hospital death following EAR through univariable and multivariable logistic regression analyses. Patients in the in-hospital death group exhibited significantly higher rates of cerebral malperfusion, mesenteric malperfusion, CPStatus, and the need for CABG compared to those in the survival group. These findings are consistent with previous research [2, 28, 29, 30, 31, 32, 33]. Malperfusion of critical organs such as the brain and heart, if not promptly relieved from ischemia, often leads to irreversible damage and postoperative complications, contributing to increased mortality rates [31, 32]. The mechanism by which organ malperfusion increases mortality is linked to the harmful cascade of inflammatory responses triggered by ischemia-reperfusion injury, resulting in metabolic acidosis and organ dysfunction [34]. In particular, the diagnosis, management, and decision-making for mesenteric malperfusion remain complex [35]. Patients with ATAAD and mesenteric malperfusion often succumb due to delays in diagnosis. The need for concurrent CABG typically indicates significant coronary malperfusion or hemodynamic instability following cardiac resuscitation [36]. These conditions compromise cardiac function, adversely affecting postoperative survival rates. Numerous studies corroborate our findings, consistently identifying cerebral malperfusion, mesenteric malperfusion, CPStatus, and the need for CABG as predictive factors for in-hospital mortality in patients with ATAAD [10, 37, 38, 39, 40].

In this study, patients in the in-hospital death group exhibited significantly higher levels of D2 and INR, as well as prolonged CPB times, compared to those in the survival group. These findings are consistent with previous research [30, 41, 42, 43, 44]. Elevated D2 levels in acute aortic dissection are strongly associated with activation of the coagulation system within the false lumen [45], reflecting a state of hypercoagulability and secondary hyperfibrinolysis [46]. Prior studies [43, 44] have demonstrated that plasma D2 concentrations correlate with factors such as vessel involvement length, dissection size, and injury characteristics. Patients with elevated D2 levels are more likely to experience organ ischemia and more extensive dissections [47]. Increased D2 levels have also been linked to reduced Plt, higher transfusion requirements during surgery, prolonged operative times, and a greater likelihood of in-hospital mortality. The prognostic significance of preoperative D2 elevation in ATAAD has been widely reported [43, 45, 48]. Preoperative elevation in INR indicates severe coagulopathy, which exacerbates bleeding tendencies. Emergency aortic repair for ATAAD presents a particularly high risk of bleeding due to prolonged CPB time, the induction of moderate to severe hypothermia, and the fragility of the dissected aorta [42]. Patients with elevated preoperative INR face a markedly increased risk of perioperative bleeding, which can lead to in-hospital mortality. Prolonged CPB time has been identified as an independent risk factor for poor outcomes following EAR for ATAAD, consistent with the findings of Macrina *et al*. [30] and Zhang *et al*. [41]. The complexity of EAR often necessitates extended CPB durations, which cannot fully replicate the body’s physiological blood supply. Prolonged CPB activates inflammatory responses, disrupts coagulation mechanisms, and causes significant damage to critical organs [48]. It may result in pulmonary dysfunction, systemic inflammatory responses, cytotoxin production, embolism, and reperfusion injury [49], all of which contribute to higher in-hospital mortality rates.

The combination of cerebral malperfusion, mesenteric malperfusion, CPStatus, D2, Plt, CABG, intraoperative blood product transfusion, and CPB time demonstrated significant predictive power for in-hospital mortality. Smith *et al*. [50] reported a link between massive plasma transfusion and adverse outcomes after cardiac surgery, likely due to complications associated with transfusion. These complications include transfusion-related acute lung injury, transfusion-associated circulatory overload, febrile and allergic reactions, infections, and multi-organ dysfunction, all of which are strongly associated with increased in-hospital mortality risk [51, 52, 53]. The prognostic importance of Plt in predicting mortality from aortic dissection has been extensively studied [54, 55, 56, 57], showing that decreased preoperative platelet levels are correlated with bleeding complications and increased fatality risk. In this study, the SVM algorithm effectively identified platelets as a significant predictor, consistent with previous findings.

This study developed a simple, effective, accurate, and practical model to predict the risk of in-hospital mortality following EAR. Our model offers several advantages over other predictive models [6, 8, 10, 11]. First, we compared multiple modeling approaches and selected the model with the best predictive performance. Second, our model incorporated a wide range of factors, including demographic characteristics, comorbidities, preoperative conditions, laboratory values, TTE data, and surgical details, making it comprehensive in assessing in-hospital mortality risk. Third, it was constructed using clinical data from a large cohort of ATAAD patients undergoing EAR at a high-volume center over an 8-year period. Lastly, we provided nomograms and a web-based calculator, enabling other researchers and clinicians to input their own data to estimate in-hospital mortality risk following EAR. This nomogram-based predictive model empowers surgeons to accurately assess the risk of in-hospital mortality for ATAAD patients undergoing EAR. As an effective visualization tool, it facilitates precise postoperative evaluations and patient risk stratification. By applying this model, surgeons can improve the quality of postoperative care, develop personalized treatment plans, and implement effective surgical strategies, ultimately enhancing survival rates and overall therapeutic outcomes.

Although some of the selected factors in this study are not novel, their combination was employed to develop a nomogram capable of predicting the risks of adverse events. However, external validation using larger sample sizes remains necessary before clinical implementation. This can be achieved through multi-center and/or multinational collaborative efforts. To promote reproducibility and support further validation, the code and model equations have been provided in this paper. By refining parameters such as regression coefficients using multi-center data, potential biases in the model could be minimized, and its predictive performance optimized. Further validation with external datasets will also enhance the model’s interpretability and reliability. To assist clinicians, an online calculator has been developed to support postoperative management.

This study has several limitations. First, it was a retrospective single-center analysis, which may have introduced selection bias. Second, procedures were performed by up to five surgeons, and variations in surgical experience and technique may have contributed to uncertainty in the results. Third, the study cohort included 84 patients aged 70 years or older, accounting for only 9% of the population, with a mortality rate of 11.9%. This suggests that the model may have limited predictive value for elderly patients. Fourth, among the cohort, 71.1% of patients had hypertension, whereas the prevalence of other comorbidities was less than 5%. As a result, the predictive value of this model for patients with multiple comorbidities may be restricted. Fifth, despite the measures taken to prevent it, machine learning algorithms may still be susceptible to overfitting. Finally, external validation with independent cohorts has yet to be conducted.

## 5. Conclusions

We developed a novel nomogram-based risk prediction model using the SVM algorithm to predict in-hospital mortality following extended aortic arch repair for ATAAD. The model demonstrated good discrimination and accuracy. The combination of cerebral malperfusion, mesenteric malperfusion, CPStatus, MFS, D2, Plt, CABG, and CPB time was identified as having significant predictive capability.

## Availability of Data and Materials

The datasets used and/or analyzed during the current study are available from the corresponding author upon reasonable request.

## Supplementary Material

Supplementary material associated with this article can be found, in the online version, at https://doi.org/10.31083/RCM26943.
