# Supplementary material for: Development and Validation of a Novel Nomogram Risk Prediction Model for In-Hospital Death Following Extended Aortic Arch Repair for Acute Type A Aortic Dissection
Source: Rev Cardiovasc Med. 2025 Apr 21;26(4):26943. doi: 10.31083/RCM26943 (PMC12059769; doi:10.31083/RCM26943)
Supplement: Supplementary file 1 [file 2153-8174-26-4-26943-s1.zip › RCM26943-Supplementary Material-V2.docx]

**Supplemental Methods**

**Contents:**

Supplementary Fig. 1. The distribution of variables before and after imputation2

Supplementary Fig. 2. The accuracy of the prediction models during the variable selection process5

Supplementary Fig. 3. The importance of variables in each machine learning model6

Supplementary Table 1 Characteristics of study population7

### Supplementary Fi. 1. The distribution of variables before and after imputation


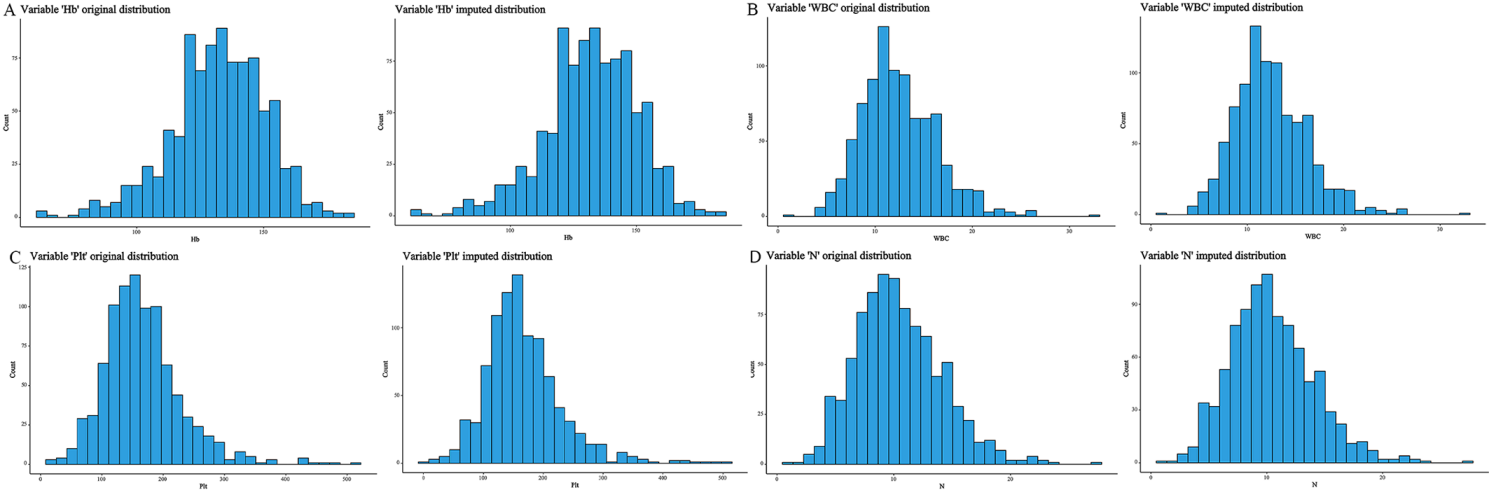


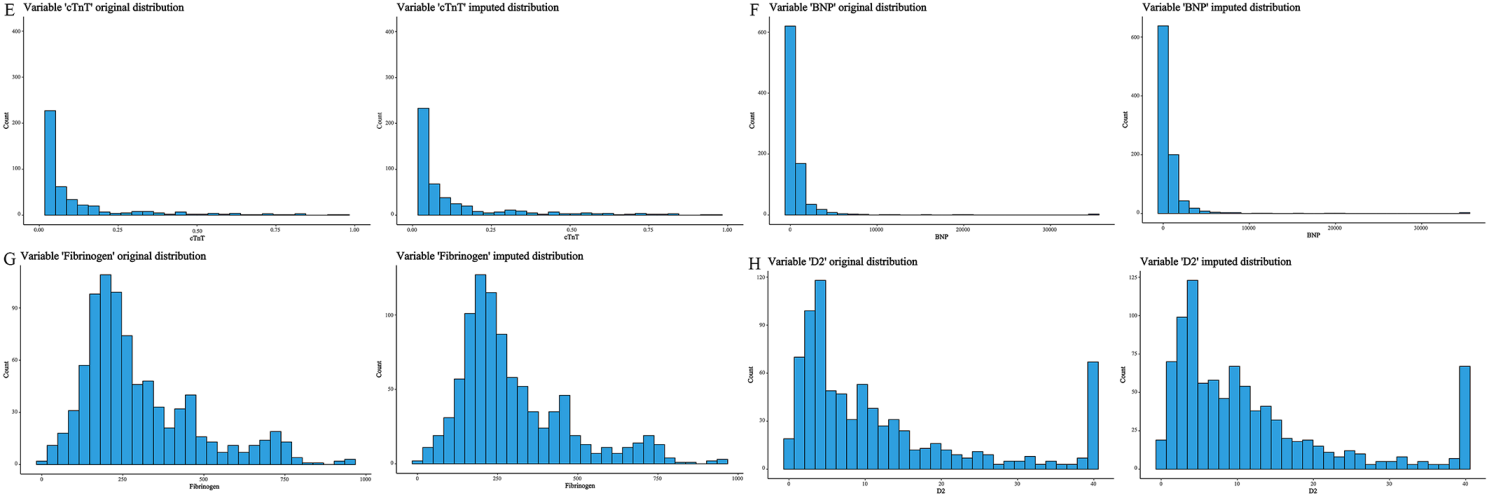


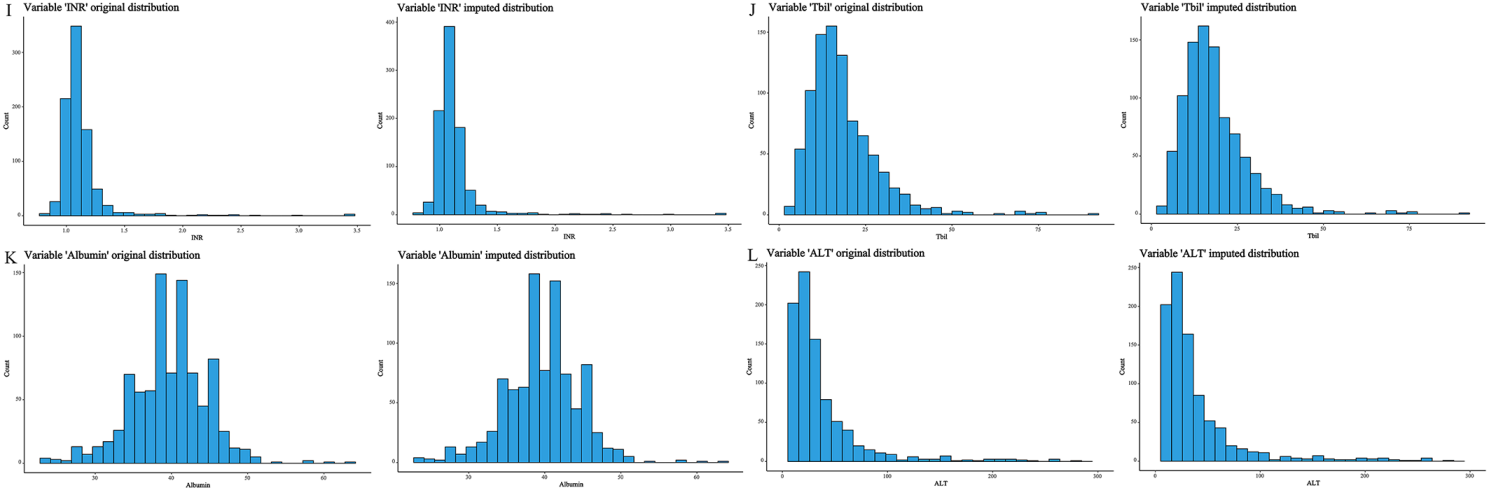


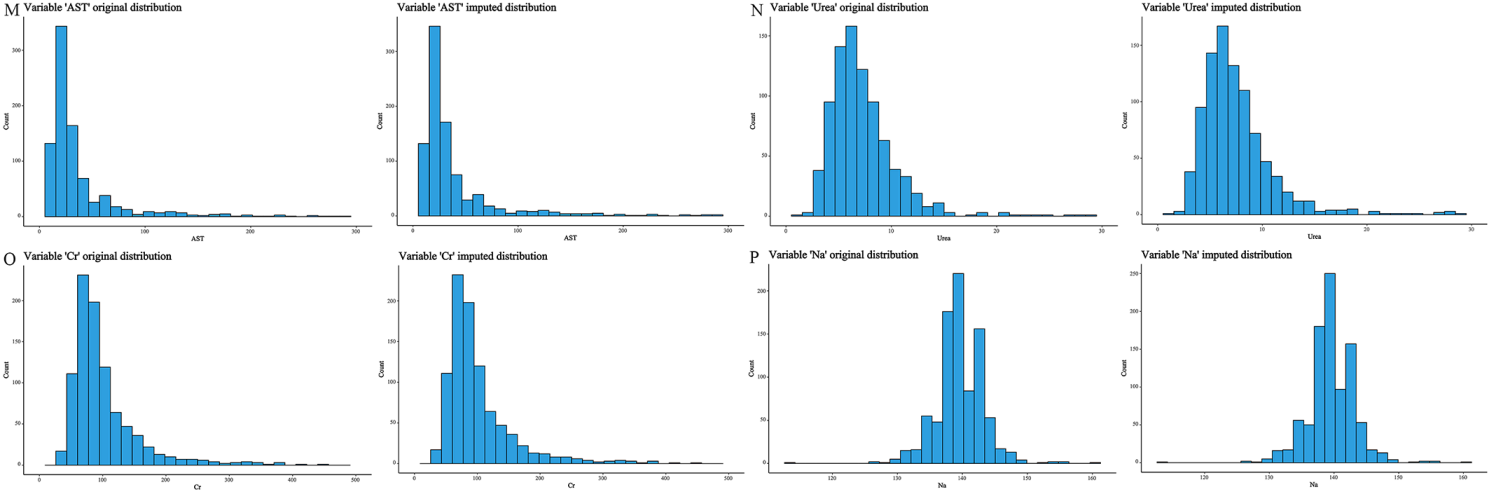


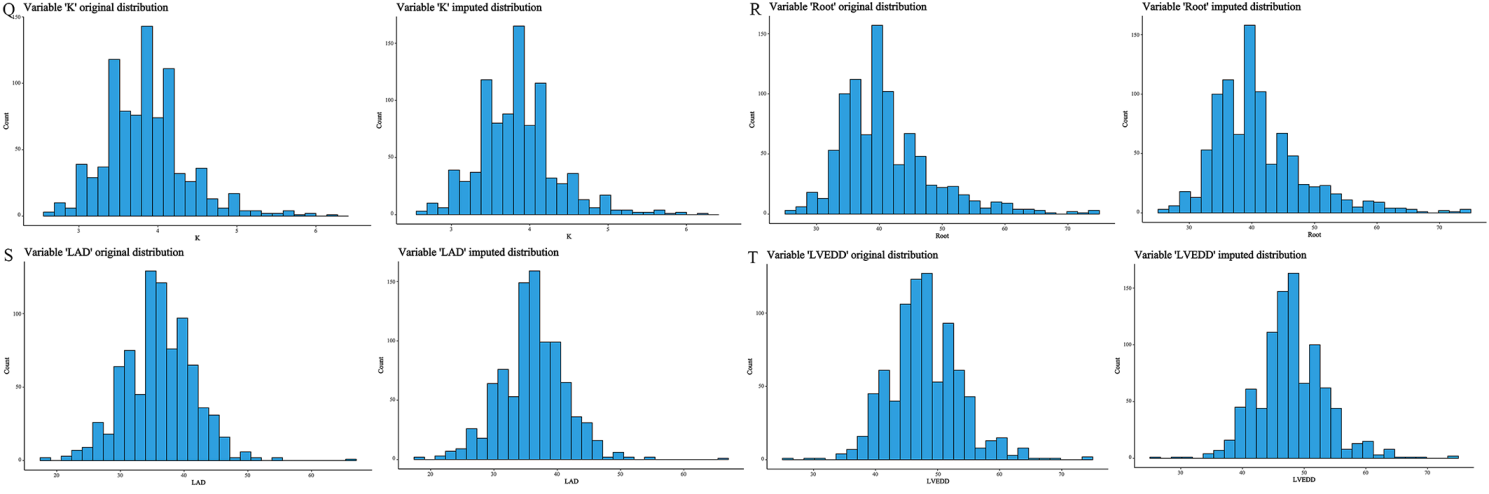


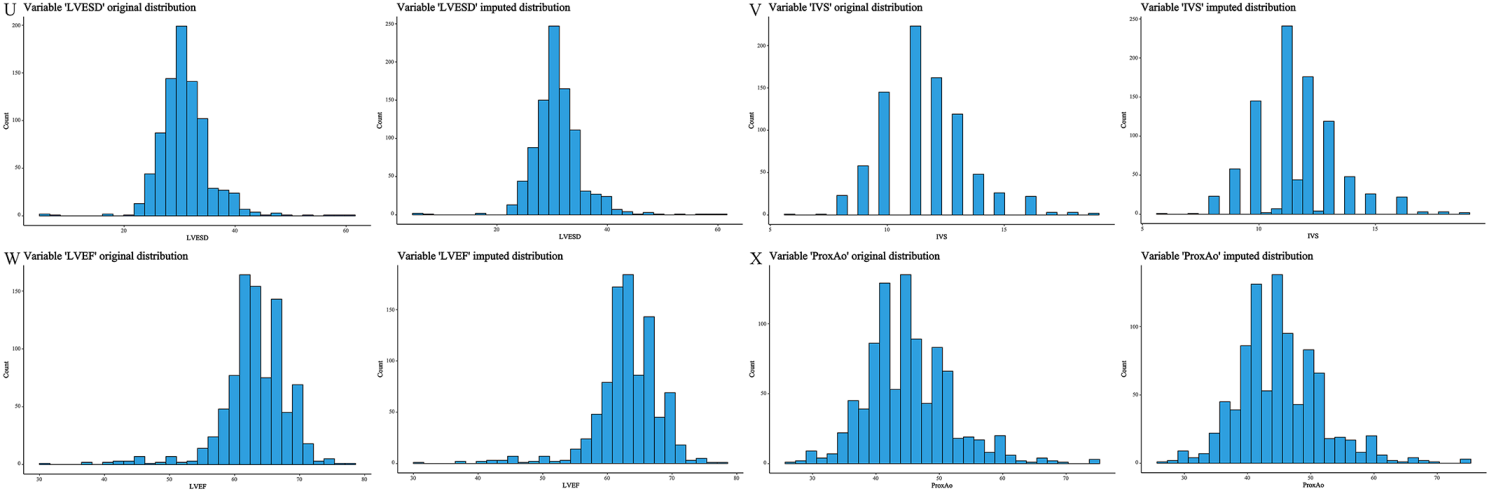


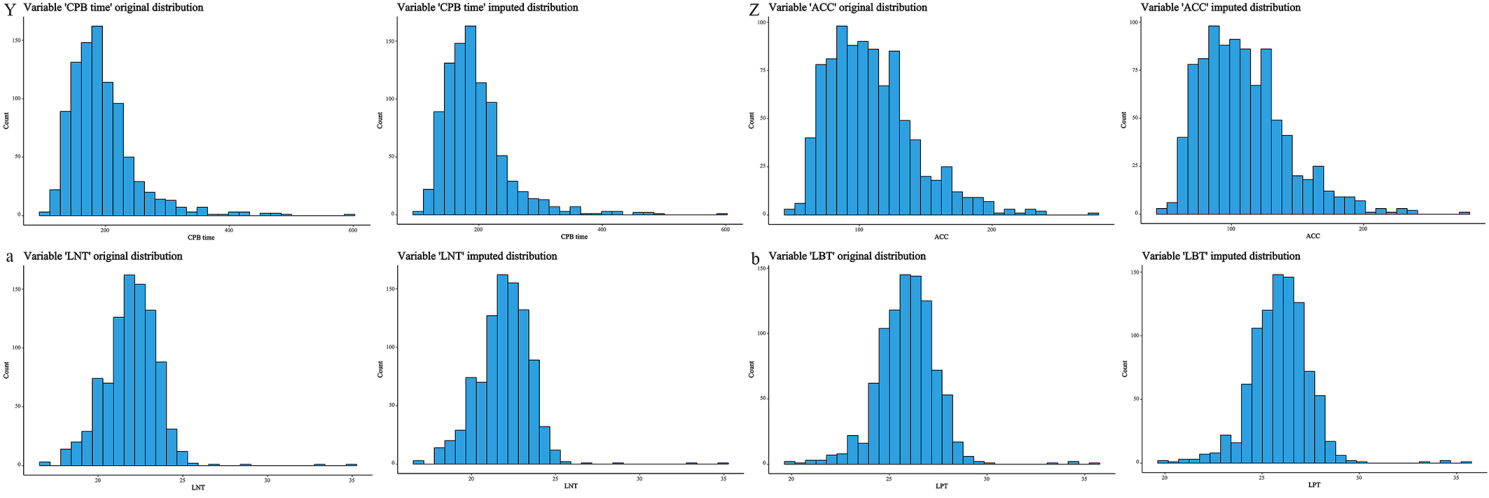


A: the distribution of Hb before and after imputation; B: the distribution of WBC before and after imputation; C: the distribution of Plt before and after imputation; D: the distribution of N before and after imputation; E: the distribution of cTnT before and after imputation; F: the distribution of BNP before and after imputation; G: the distribution of Fibrinogen before and after imputation; H: the distribution of D2 before and after imputation; I: the distribution of INR before and after imputation; J: the distribution of Tbil before and after imputation; K: the distribution of Albumin before and after imputation; L: the distribution of ALT before and after imputation; M: the distribution of AST before and after imputation; N: the distribution of Urea before and after imputation; O: the distribution of Cr before and after imputation; P: the distribution of Na before and after imputation; Q: the distribution of K before and after imputation; R: the distribution of Root before and after imputation; S: the distribution of LAD before and after imputation; T: the distribution of LVEDD before and after imputation; U: the distribution of LVESD before and after imputation; V: the distribution of IVS before and after imputation; W: the distribution of LVEF before and after imputation; X: the distribution of ProxAo before and after imputation; Y: the distribution of CPB time before and after imputation; Z: the distribution of ACC before and after imputation; a: the distribution of LNT before and after imputation; b: the distribution of LBT before and after imputation.

Hb, hemoglobin, g/L; WBC, white blood cell count, x10^12/L; Plt, platelet count, x10^9/L; N, neutrophil count, x10^12/L; cTnT, cardiac troponin T, ng/mL; BNP, n-terminal pro-brain natriuretic peptide, pg/mL; D2, D-dimer, mg/L; INR, international normalized ratio; ALT, alanine transaminase, U/L; AST, aspartate aminotransferase, U/L; Cr, creatinine levels, umol/L; LAD, diameter of left atrium, mm; LVEDD, left ventricular end-diastolic dimension, mm; LVESD, left ventricular end-diastolic dimension, mm; IVS, interventricular septum, mm; ProxAo, diameter of proximal aortic artery, mm; CPB, cardiopulmonary bypass, min; ACC, aortic cross-clamp time, min; LNT, lowest nose temperature, ℃; LPT, lowest bladder temperature, ℃.

### Supplementary Fig. 2. The accuracy of the prediction models during the variable selection process


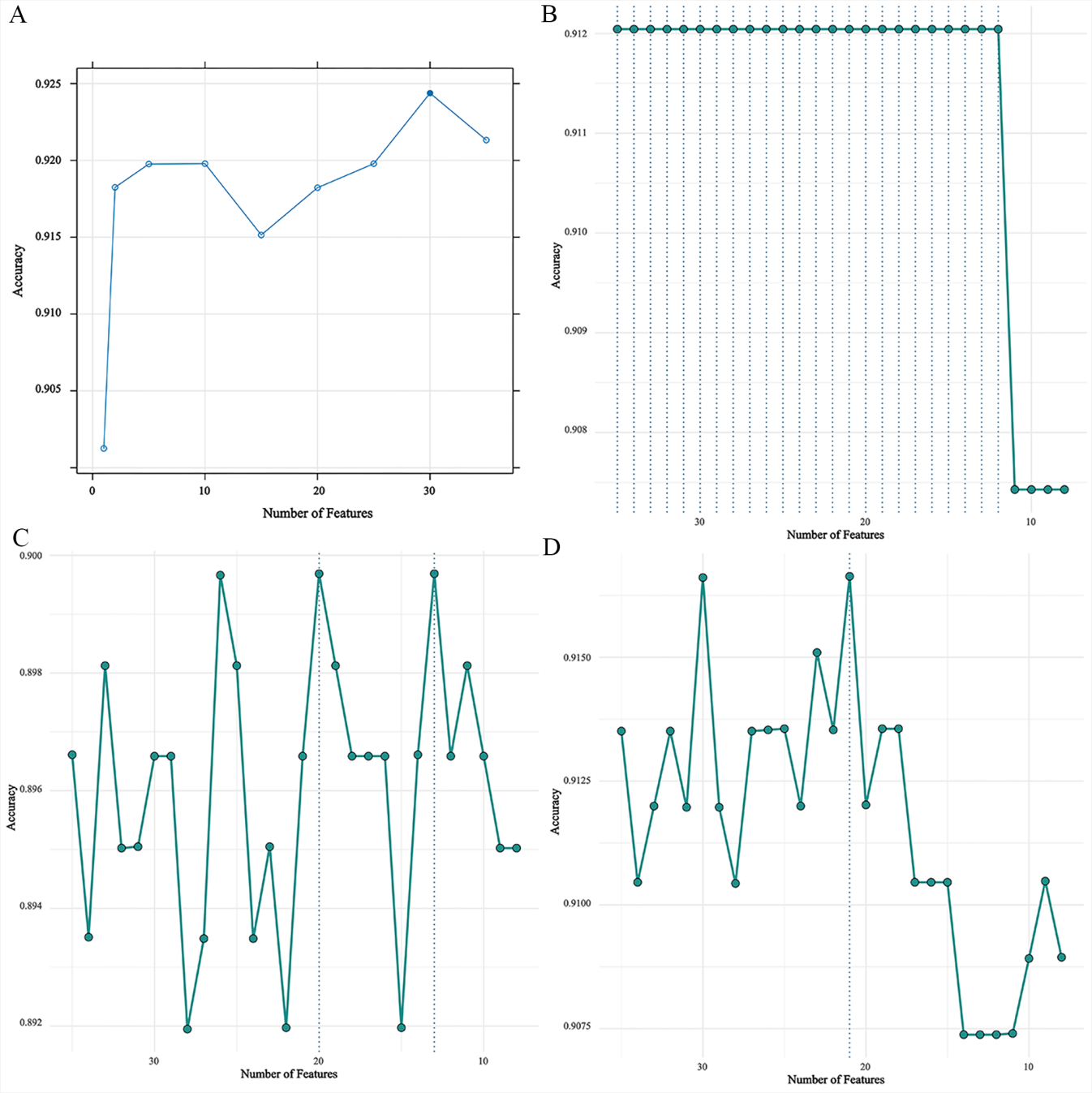


A: the variable selection process in RF model; B: the variable selection process in Dtree model; C: the variable selection process in XGBoost model; D:the variable selection process in SVM model.

### Supplementary Fig. 3. The importance of variables in each machine learning model


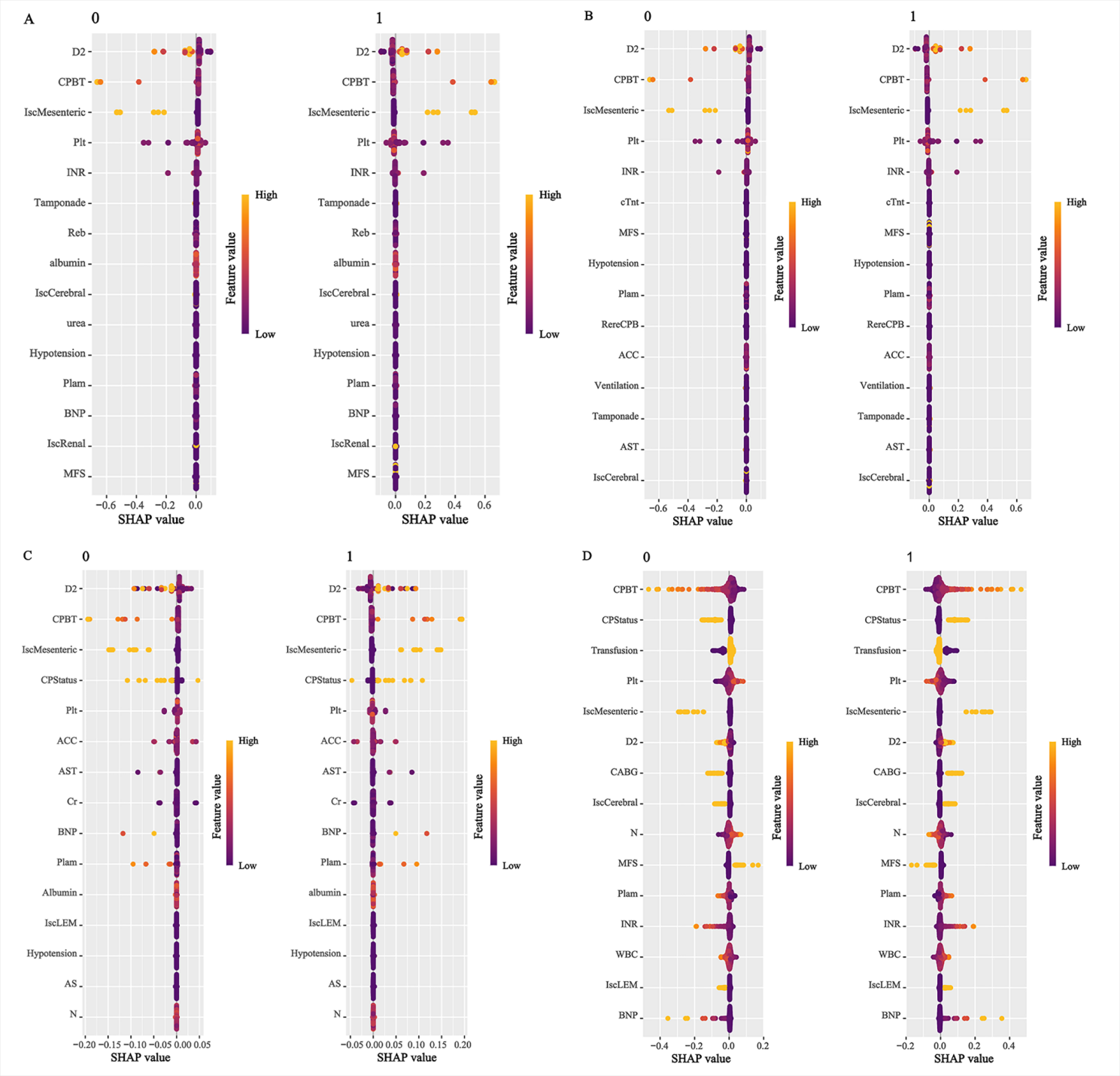


A: the importance of variables in RF model; B: the importance of variables in Dtree model; C: the importance of variables in XGBoost model; D: the importance of variables in SVM model.

IscCerebral, cerebral malperfusion; IscMesenteric, mesenteric malperfusion; IscLEM, lower extremity malperfusion; WBC, white blood cell count; Plt, platelet count; N, neutrophil count; BNP, n-terminal pro-brain natriuretic peptide; D2, D-dimer; INR, international normalized ratio; Cr, creatinine levels; AST, aspartate aminotransferase; CABG, coronary artery bypass grafting; Plam, intraoperative plasma transfusions; CPB, cardiopulmonary bypass; CPBT, cardiopulmonary bypass time; ACC, aortic cross-clamp time; CPStatus, preoperative critical station; MFS, Marfan syndrome; cTnt, cardiac troponin T.

**Supplementary Table 1 Characteristics of study population**

| Variable | Death  (N=84) | Survival  (N=841) | p |
| --- | --- | --- | --- |
| **Demographics** |  |  |  |
| Male | 73(86.9%) | 658(78.2%) | 0.063 |
| Age, years | 57.0(44.0-64.0) | 52.0(43.0-62.0) | 0.084 |
| Height, cm | 170.5(167.3-175.0) | 170.0(165.0-175.0) | 0.557 |
| Weight, kg | 75.0(65.0-83.0) | 75.0(65.0-83.0) | 0.886 |
| BMI, kg/m2 | 25.0(22.6-28.0) | 25.6(23.2-27.8) | 0.428 |
| Somking | 12(14.3%) | 156(18.5%) | 0.334 |
| **Comorbidity** |  |  |  |
| HBP | 64(76.2%) | 594(70.6%) | 0.284 |
| DM | 7(8.3%) | 34(4.0%) | 0.123 |
| Stroke | 6(7.1%) | 33(3.9%) | 0.265 |
| CAD | 5(6.0%) | 35(4.2%) | 0.626 |
| CKD | 2(2.4%) | 15(1.8%) | >0.999 |
| AF | 2(2.4%) | 11(1.3%) | 0.756 |
| COPD | 0 | 7(0.8%) | >0.999 |
| AD family history | 0 | 22(2.6%) | 0.261 |
| BAV | 4(4.8%) | 22(2.6%) | 0.430 |
| MFS | 2(2.4%) | 75(8.9%) | 0.039 |
| Heart surgery history | 6(7.1%) | 28(3.3%) | 0.142 |
| Previous TEVAR | 0 | 27(3.2%) | 0.185 |
| **Medical history** |  |  |  |
| Anticoagulation drugs | 3(3.6%) | 11(1.3%) | 0.127 |
| Warfarin sodium | 3(3.6%) | 9(1.1%) | 0.087 |
| Rivaroxaban | 0 | 2(0.2%) | >0.999 |
| Antiplatelet drugs | 5(6.0%) | 24(2.9%) | 0.175 |
| aspirin | 5(6.0%) | 24(2.9%) | 0.175 |
| clopidogrel | 1(1.2%) | 8(1.0%) | 0.577 |
| ticagrelor | 0 | 0 | >0.999 |
| **Malperfusion** |  |  |  |
| IscCoronary | 11(13.1%) | 23(2.7%) | <0.001 |
| IscCerebral | 20(23.8%) | 71(8.4%) | <0.001 |
| IscSpinal | 7(8.3%) | 11(1.3%) | <0.001 |
| IscMesenteric | 11(13.1%) | 13(1.5%) | <0.001 |
| IscRenal | 13(15.5%) | 60(7.1%) | 0.007 |
| IscUEM | 3(3.6%) | 10(1.2%) | 0.200 |
| IscLEM | 20(23.8%) | 87(10.3%) | <0.001 |
| **Critical preoperative status** | 31(36.9%) | 62(7.4%) | <0.001 |
| Hypotension | 11(13.1%) | 25(3.0%) | <0.001 |
| Shock | 3(3.6%) | 3(0.4%) | 0.012 |
| Tamponade | 10(11.9%) | 23(2.7%) | <0.001 |
| Ventilation | 11(13.1%) | 21(2.5%) | <0.001 |
| **Laboratory data** |  |  |  |
| Hb, g/L | 133.5(124.0-147.0) | 133.0(121.0-144.3) | 0.502 |
| WBC, x10^12/L | 13.1(10.7-16.3) | 11.9(9.8-14.6) | 0.016 |
| Plt, x10^9/L | 141.0(113.0-186.3) | 158.0(129.0-197.0) | 0.002 |
| N, x10^12/L | 11.5(8.3-14.2) | 10.1(7.9-12.5) | 0.026 |
| cTnT, x1000ng/mL | 47.5(14.5-137.3) | 20.0(9.0-66.0) | <0.001 |
| BNP, pg/mL | 400.4(209.9-1397.8) | 321.0(146.5-739.4) | 0.011 |
| Fibrinogen, mg/dL | 195.9(161.3-321.0) | 248.0(188.2-383.0) | 0.002 |
| D2, mg/L | 15.6(7.5-26.6) | 8.2(3.9-14.4) | <0.001 |
| INR | 1.1(1.1-1.2) | 1.1(1.0-1.1) | <0.001 |
| Tbil, umol/L | 14.4(11.6-21.8) | 16.5(12.3-22.6) | 0.208 |
| Albumin, g/L | 38.0(36.0-41.1) | 40.0(37.0-43.0) | 0.011 |
| ALT, U/L | 29.0(17.0-65.8) | 26.0(16.0-44.0) | 0.134 |
| AST, U/L | 32.5(21.0-101.5) | 25.0(18.0-40.0) | 0.001 |
| Urea, mmol/L | 7.9(6.2-11.4) | 6.7(5.3-8.6) | <0.001 |
| Cr, umol/L | 109.0(80.0-149.8) | 84.0(68.0-111.5) | <0.001 |
| Na, mmol/L | 140.0(137.8-141.0) | 139.9(138.0-142.0) | 0.479 |
| K, mmol/L | 3.9(3.6-4.3) | 3.8(3.5-4.1) | 0.060 |
| **TTE data** |  |  |  |
| Root, mm | 39.0(37.0-43.8) | 40.0(36.0-44.0) | 0.849 |
| LAD, mm | 37.0(34.0-39.7) | 36.0(33.0-49.0) | 0.368 |
| LVEDD, mm | 47.0(44.0-51.3) | 48.0(45.0-51.0) | 0.606 |
| LVESD, mm | 31.0(29.0-33.0) | 30.7(29.0-33.0) | 0.989 |
| IVS, mm | 12.0(11.0-13.0) | 11.0(10.0-12.4) | 0.007 |
| LVEF, % | 63.0(60.0-66.0) | 63.7(61.0-66.0) | 0.236 |
| ProxAo, mm | 45.0(41.0-49.0) | 44.7(41.0-49.0) | 0.848 |
| AI |  |  | 0.145 |
| no or trace | 25(29.8%) | 334(39.7%) |  |
| mild | 26(31.0%) | 203(24.1%) |  |
| moderate | 14(16.7%) | 177(21.0%) |  |
| moderate to sever | 12(14.3%) | 81(9.6%) |  |
| severe | 7(8.3%) | 46(5.5%) |  |
| AS |  |  | 0.005 |
| no or trace | 82(97.6%) | 834(99.2%) |  |
| mild | 1(1.2%) | 2(0.2%) |  |
| moderate | 0 | 5(0.6%) |  |
| moderate to sever | 1(1.2%) | 0 |  |
| severe | 0 | 0 |  |
| Pericardial effusion | 30(35.7%) | 269(30.9%) | 0.366 |
| Characteristics of dissection |  |  |  |
| IMH | 6(7.1%) | 92(10.9%) | 0.281 |
| PAU | 1(1.2%) | 19(2.3%) | 0.804 |
| Thrombosis of the false lumen |  |  |  |
| Root | 2(2.4%) | 93(11.1%) | 0.012 |
| Ascending | 18(21.4%) | 210(25.0%) | 0.473 |
| Arch | 9(10.7%) | 89(10.6%) | 0.970 |
| Descending | 1(1.2%) | 19(2.3%) | 0.521 |
| Entry tear |  |  |  |
| Root | 3(3.6%) | 29(3.4%) | >0.999 |
| Ascending | 43(51.2%) | 419(49.8%) | 0.811 |
| Arch | 30(35.7%) | 283(33.7%) | 0.703 |
| Descending | 18(21.4%) | 199(23.7%) | 0.645 |
| Commissure detachment | 32(38.1%) | 401(47.7%) | 0.093 |
| Sinus involved | 51(60.7%) | 556(66.1%) | 0.321 |
| Coronary involvement |  |  |  |
| RCA |  |  | 0.088 |
| None | 61(72.6%) | 628(74.7%) |  |
| Neri A | 4(4.8%) | 72(8.6%) |  |
| Neri B | 14(16.7%) | 124(14.7%) |  |
| Neri C | 5(6.0%) | 17(2.0%) |  |
| LCA |  |  | 0.168 |
| None | 79(94.0%) | 802(95.4%) |  |
| Neri A | 0 | 16(1.9%) |  |
| Neri B | 5(6.0%) | 21(2.5%) |  |
| Neri C | 0 | 0 |  |
| Supra-aortic vessels involvement |  |  |  |
| IA | 55(65.5%) | 535(63.6%) | 0.735 |
| LCCA | 43(51.2%) | 386(45.9%) | 0.354 |
| LSCA | 43(51.2%) | 375(44.5%) | 0.246 |
| Duration |  |  |  |
| Sym.hosT, h | 14.0(7.0-24.0) | 13.0(8.0-24.0) | 0.322 |
| Hos.surgT, h | 16.0(6.3-24.0) | 14.0(5.0-24.0) | 0.494 |
| Sym.surgT, h | 28.5(18.8-56.0) | 29.0(18.0-68.0) | 0.898 |
| Emergency | 63(75.0%) | 653(77.6%) | 0.580 |
| Surgical data |  |  |  |
| Proximal |  |  |  |
| ARR | 57(67.9%) | 575(68.4%) | 0.923 |
| Bentall | 13(15.5%) | 91(10.8%) | 0.198 |
| David | 5(6.0%) | 86(10.2%) | 0.210 |
| Wheat | 1(1.2%) | 4(0.5%) | 0.379 |
| Distal |  |  |  |
| TAR | 84(100%) | 841(100%) | >0.999 |
| FET | 83(98.8%) | 825(98.1%) | 0.970 |
| Associated surgeries |  |  |  |
| CABG | 20(23.8%) | 48(5.7%) | <0.001 |
| Other |  |  | / |
| MV procedures | 0 | 5(0.6%) | >0.999 |
| TV procedures | 0 | 0 | >0.999 |
| Perfusion |  |  |  |
| CPB time, min | 212.5(178.3-276.0) | 183.0(159.0-213.5) | <0.001 |
| Re-CPB | 3(3.6%) | 21(2.5%) | 0.818 |
| Re-re-CPB | 3(3.6%) | 1(0.1%) | 0.003 |
| ACC, min | 115.0(82.5-145.8) | 104.0(85.0-126.0) | 0.043 |
| Re-ACC | 3(3.6%) | 9(1.1%) | 0.154 |
| DHCA, min | 21.0(17.0-26.8) | 21.0(17.0-26.0) | 0.588 |
| Unilateral ACP | 82(97.6%) | 835(99.3%) | 0.159 |
| Bilateral ACP | 5(6.0%) | 16(1.9%) | 0.046 |
| LNT, ℃ | 22.0(20.8-22.3) | 22.0(21.0-23.0) | 0.214 |
| LBT, ℃ | 25.8(24.8-26.8) | 26.0(25.1-26.9) | 0.434 |
| Blood product |  |  |  |
| Transfusion rate | 78(92.9%) | 645(76.7%) | 0.001 |
| Red cell, U | 7.5(4.0-10.0) | 4.0(2.0-6.0) | <0.001 |
| Plam, ml | 800.0(600.0-1200.0) | 600.0(5.0-800.0) | <0.001 |

BMI, body mass index; HBP, hypertension; DM, Diabetes mellitus; CAD, coronary artery disease; CKD, chronic kidney disease; COPD, chronic obstructive pulmonary disease; AD, aortic dissection; MFS, Marfan syndrome; BAV, bicuspid aortic valve; TEVAR, thoracic endovascular aortic repair; IscCerebral, cerebral malperfusion; IscSpinal, spinal malperfusion; IscCoronary, coronary malperfusion; IscMesenteric, mesenteric malperfusion; IscRenal, renal malperfusion; IscUEM, upper extremity malperfusion; IscLEM, lower extremity malperfusion; Hb, hemoglobin; WBC, white blood cell count; Plt, platelet count; N, neutrophil count; BNP, n-terminal pro-brain natriuretic peptide; D2, D-dimer; INR, international normalized ratio; ALT, alanine transaminase; AST, aspartate aminotransferase; Cr, creatinine levels; LAD, diameter of left atrium; LVEDD, left ventricular end-diastolic dimension; LVESD, left ventricular end-diastolic dimension; IVS, interventricular septum; ProxAo, diameter of proximal aortic artery; AI, aortic insufficiency; AS, aortic valve stenosis; Sym.hosT, time to hospital from symptom onset; Hos.surgT, time to surgery from hospital onset; Sym.surgT, time to surgery from symptom onset; RCA, right coronary artery; LCA, left coronary artery; IA, innominate artery; LCCA, left common carotid artery; LSCA, left subclavian artery; ARR, ascending aorta replacement with commissure resuspension; CABG, coronary artery bypass grafting; MV procedure, mitral valve procedures; TV procedures, tricuspid valve procedures; FET, frozen elephant trunk; CPB, cardiopulmonary bypass; ACC, aortic cross-clamp time; DHCA, deep hypothermic circulatory arrest; ACP, anterograde cerebral perfusion; LNT, lowest nose temperature; LPT, lowest bladder temperature; Reb, intraoperative red blood cell transfusions; Plam, intraoperative plasma transfusions.
