# Supplementary figures and images for: Development and Validation of a Novel Nomogram Risk Prediction Model for In-Hospital Death Following Extended Aortic Arch Repair for Acute Type A Aortic Dissection
Source: Rev Cardiovasc Med. 2025 Apr 21;26(4):26943. doi: 10.31083/RCM26943 (PMC12059769; doi:10.31083/RCM26943)

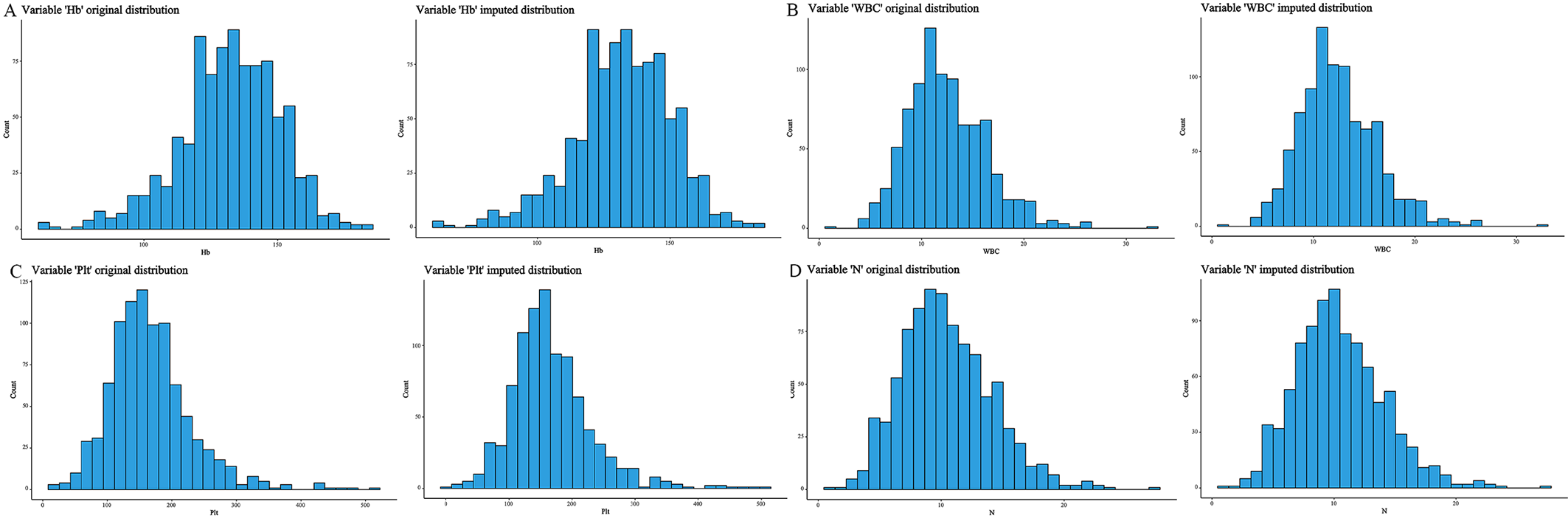

Supplement: Supplementary file 1 [file 2153-8174-26-4-26943-s1.zip › SFigure 1-1.tif]

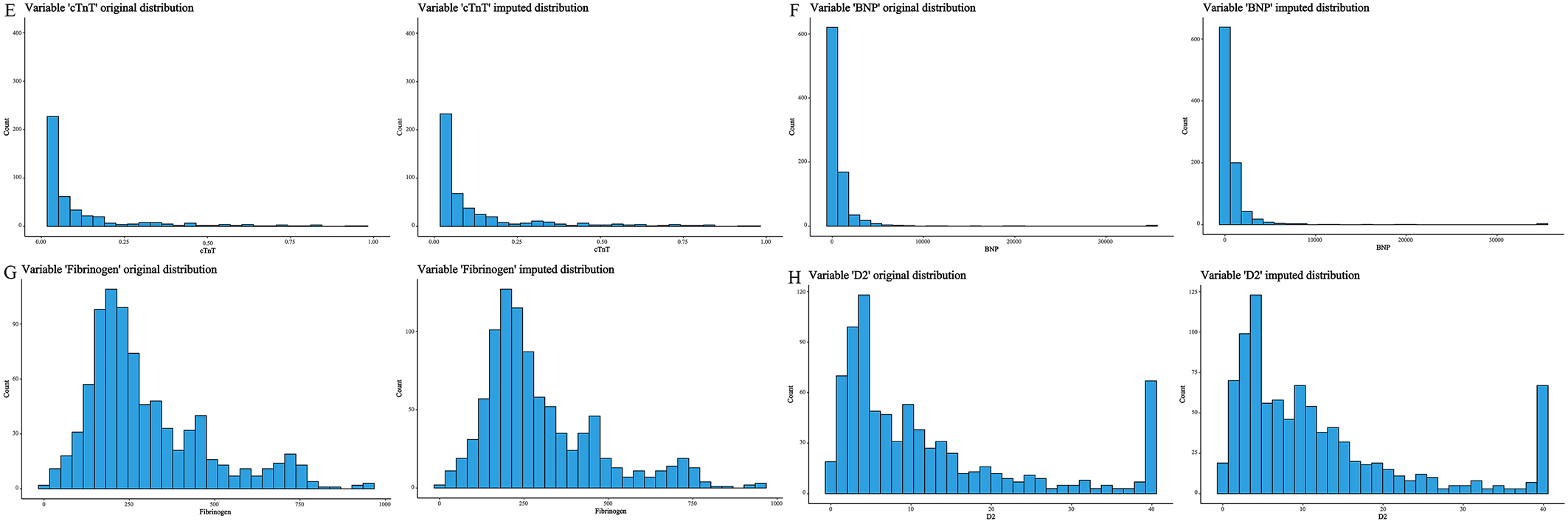

Supplement: Supplementary file 1 [file 2153-8174-26-4-26943-s1.zip › SFigure 1-2.tif]

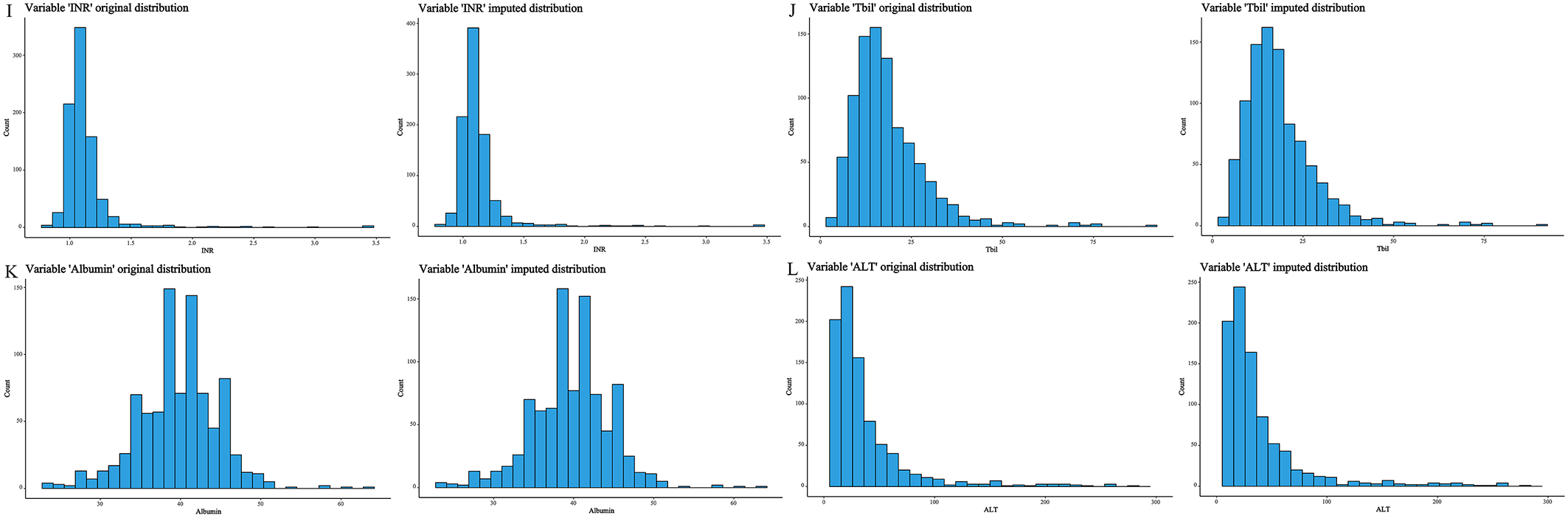

Supplement: Supplementary file 1 [file 2153-8174-26-4-26943-s1.zip › SFigure 1-3.tif]

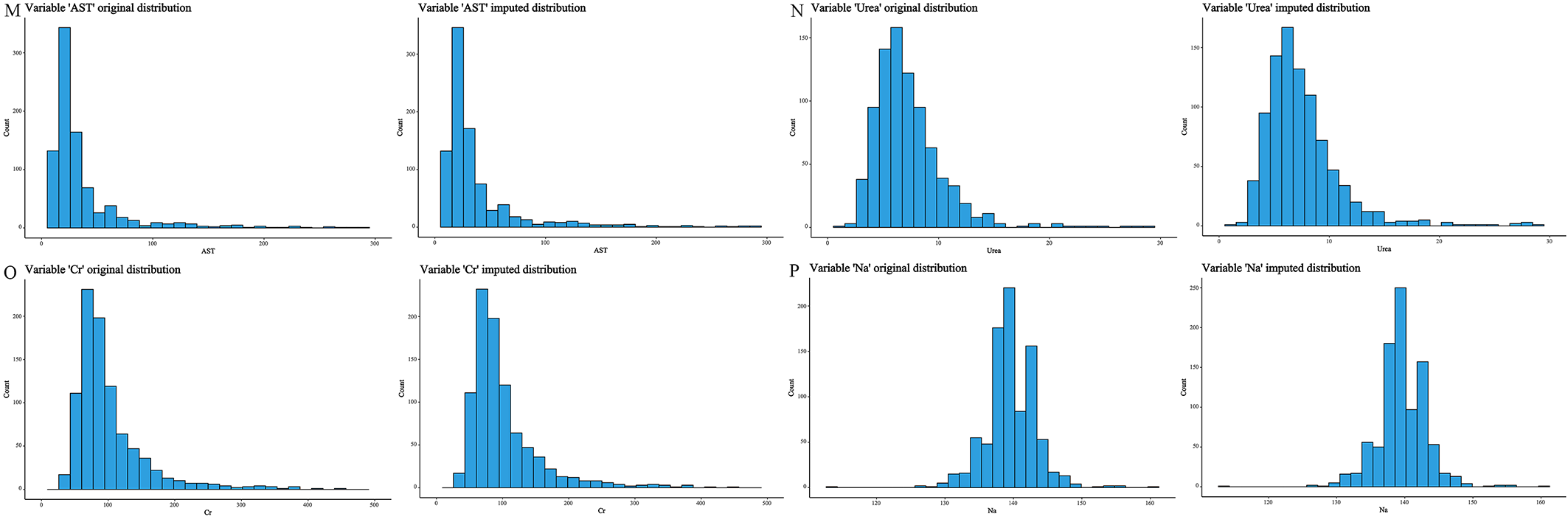

Supplement: Supplementary file 1 [file 2153-8174-26-4-26943-s1.zip › SFigure 1-4.tif]

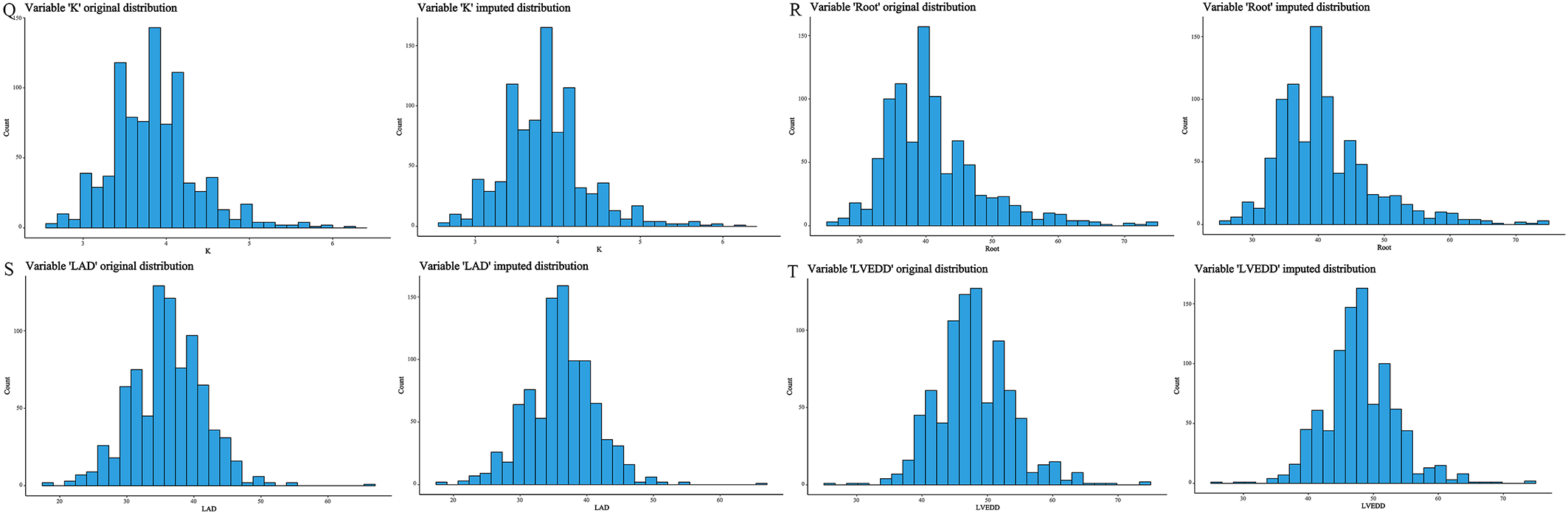

Supplement: Supplementary file 1 [file 2153-8174-26-4-26943-s1.zip › SFigure 1-5.tif]

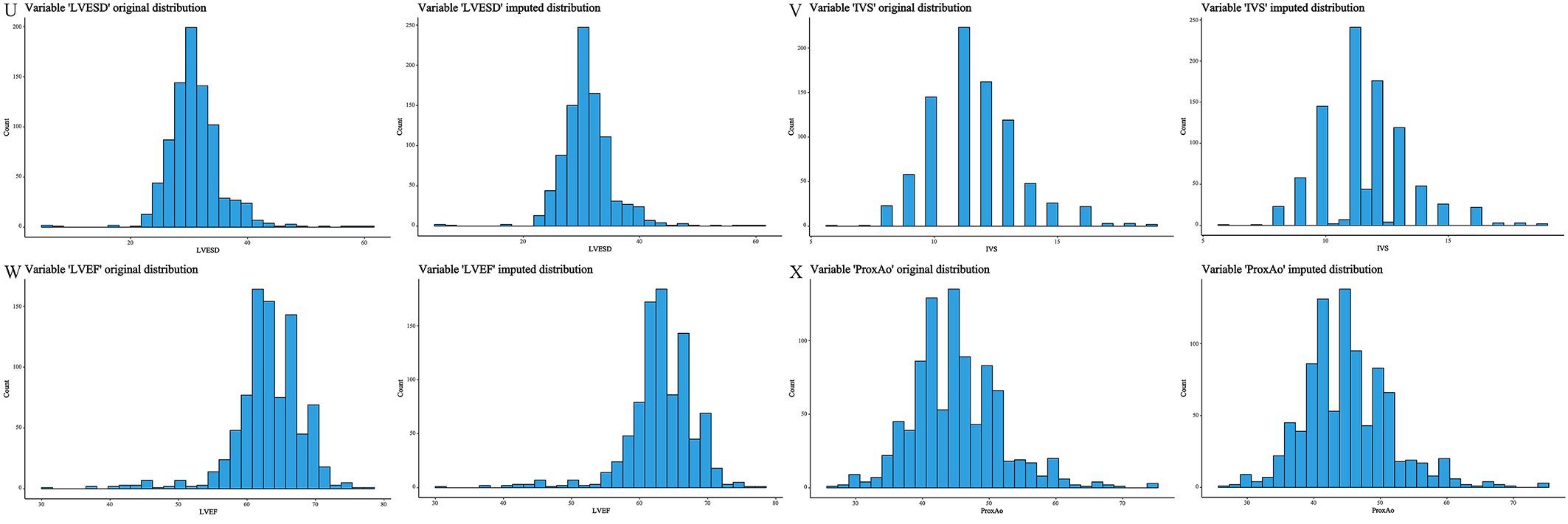

Supplement: Supplementary file 1 [file 2153-8174-26-4-26943-s1.zip › SFigure 1-6.tif]

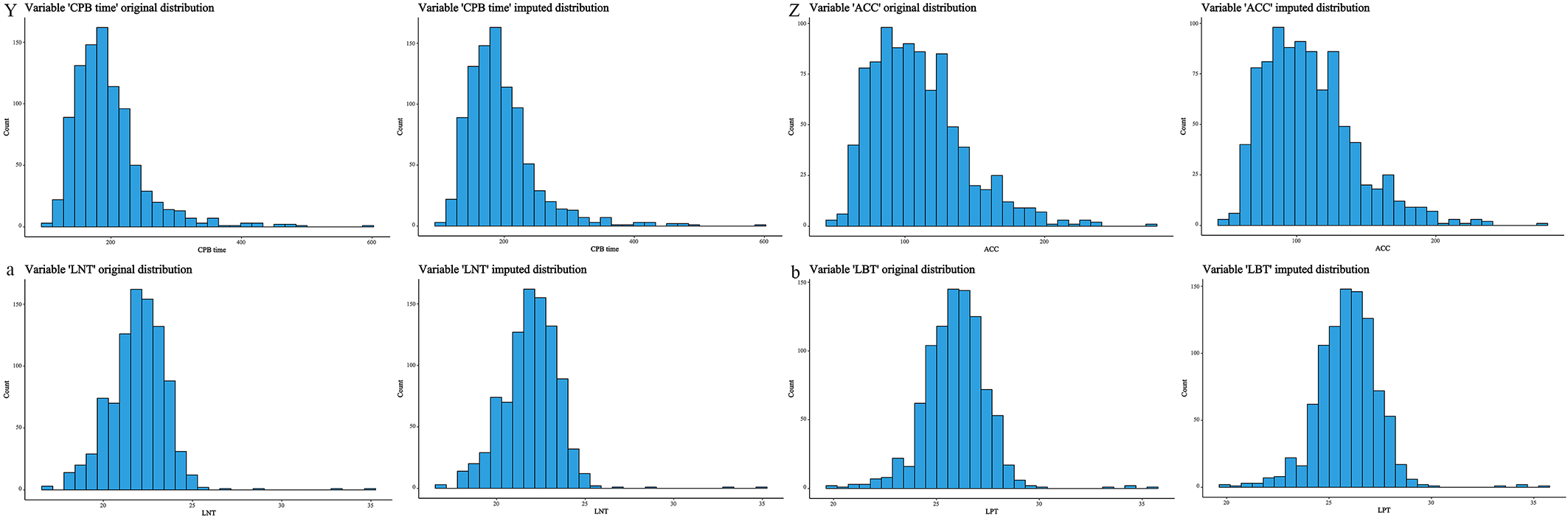

Supplement: Supplementary file 1 [file 2153-8174-26-4-26943-s1.zip › SFigure 1-7.tif]

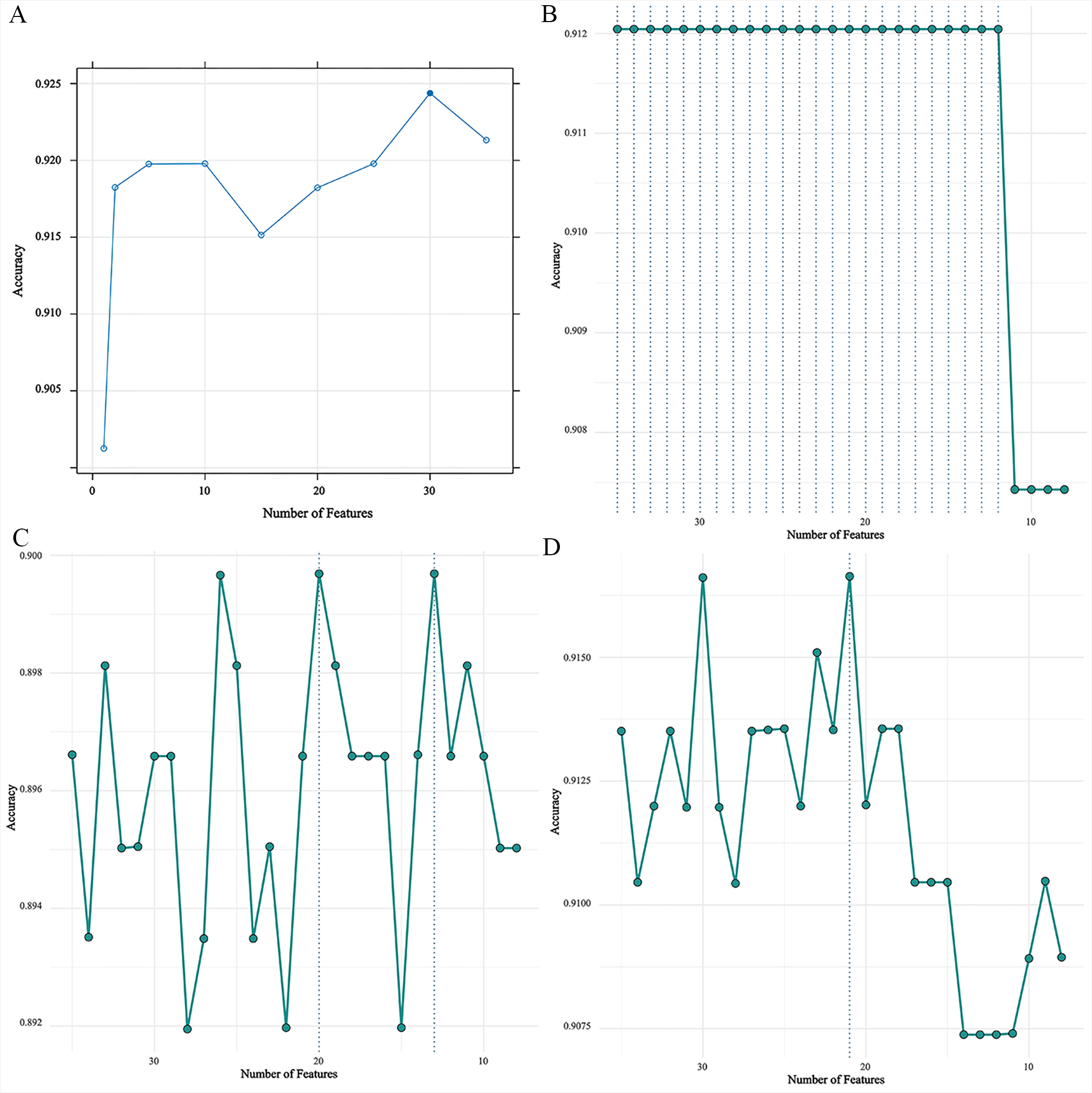

Supplement: Supplementary file 1 [file 2153-8174-26-4-26943-s1.zip › SFigure 2.tif]

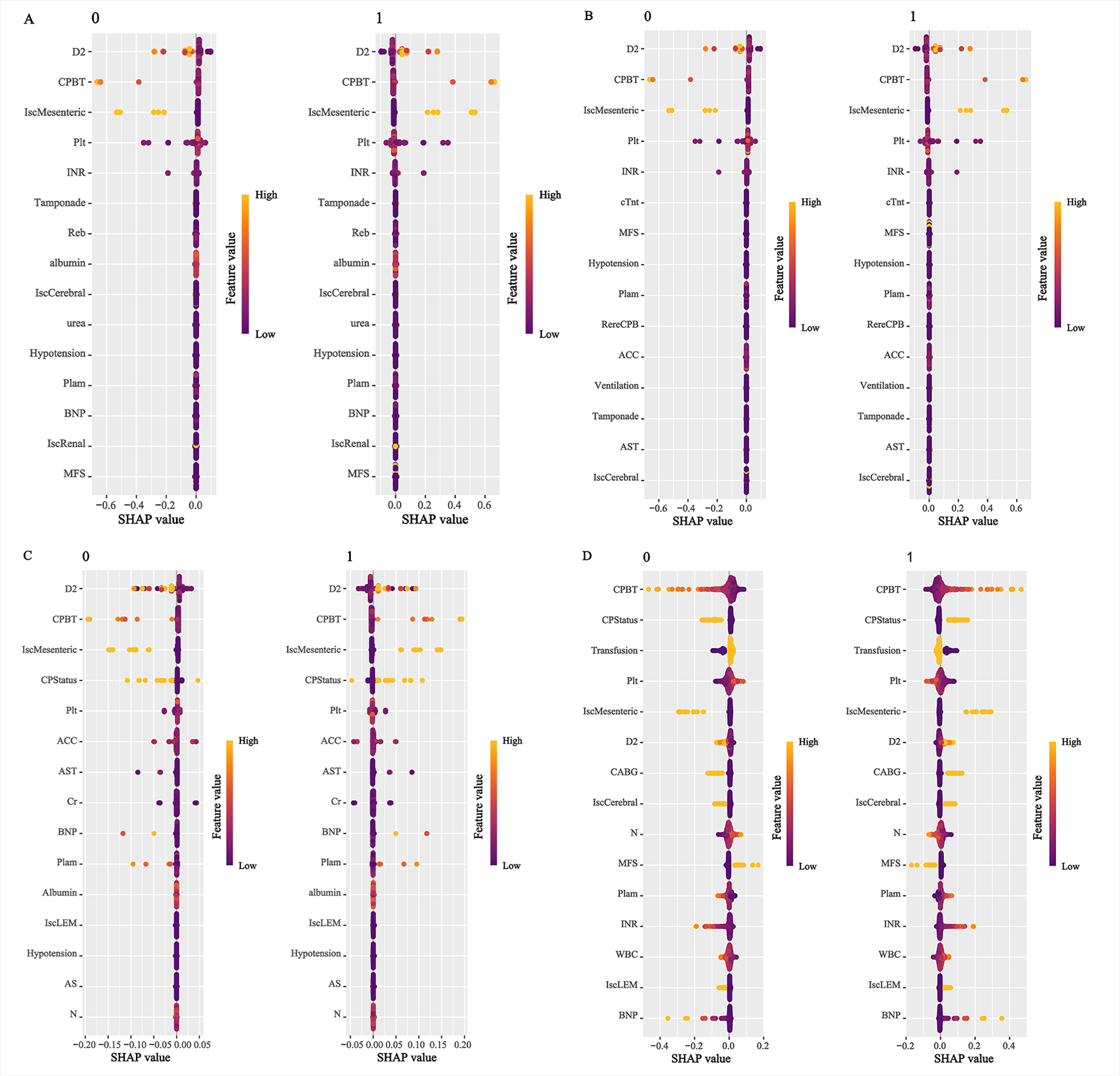

Supplement: Supplementary file 1 [file 2153-8174-26-4-26943-s1.zip › SFigure 3.tif]
